# Large‐Area Field Potential Imaging Having Single Neuron Resolution Using 236 880 Electrodes CMOS‐MEA Technology

**DOI:** 10.1002/advs.202207732

**Published:** 2023-04-23

**Authors:** Ikuro Suzuki, Naoki Matsuda, Xiaobo Han, Shuhei Noji, Mikako Shibata, Nami Nagafuku, Yuto Ishibashi

**Affiliations:** ^1^ Department of Electronics Graduate School of Engineering Tohoku Institute of Technology 35‐1 Yagiyama Kasumicho, Taihaku‐ku Sendai Miyagi 982‐8577 Japan

**Keywords:** brain organoid, brain slice, CMOS‐MEA, field potential imaging, hiPSC‐derived neuron, sensory neuron, single‐cell resolution

## Abstract

The electrophysiological technology having a high spatiotemporal resolution at the single‐cell level and noninvasive measurements of large areas provide insights on underlying neuronal function. Here, a complementary metal‐oxide semiconductor (CMOS)‐microelectrode array (MEA) is used that uses 236 880 electrodes each with an electrode size of 11.22 × 11.22 µm and 236 880 covering a wide area of 5.5 × 5.9 mm in presenting a detailed and single‐cell‐level neural activity analysis platform for brain slices, human iPS cell‐derived cortical networks, peripheral neurons, and human brain organoids. Propagation pattern characteristics between brain regions changes the synaptic propagation into compounds based on single‐cell time‐series patterns, classification based on single DRG neuron firing patterns and compound responses, axonal conduction characteristics and changes to anticancer drugs, and network activities and transition to compounds in brain organoids are extracted. This detailed analysis of neural activity at the single‐cell level using the CMOS‐MEA provides a new understanding of the basic mechanisms of brain circuits in vitro and ex vivo, on human neurological diseases for drug discovery, and compound toxicity assessment.

## Graphical abstract

Field potential imaging using a complementary metal‐oxide semiconductor (CMOS)‐microelectrode array (MEA) with 236 880 electrodes covering a wide area of 5.5 × 5.9 mm provides a single‐cell‐level evaluating the platform for detailed neural activity analysis including axonal conduction characteristics and propagation pattern characteristics in cultured neurons, brain organoids, and brain slices.

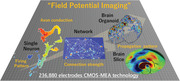

## 1 Introduction

The function of the nervous system is represented by an electrical activity. The technology for measuring the electrical activity of the nervous system is essential for understanding higher functions and neurological diseases, drug discovery development, and toxicity evaluation of compounds. The methods for measuring electrical activity include electrical measurement, optical measurement, and magnetic field measurement. Electrical measurement has a high temporal resolution, and optical measurement has a high spatial resolution. In the recent years, the density of microelectrode array (MEA) has increased, and the spatial resolution in electrical activity measurement has increased as well. With the latest advances in complementary metal‐oxide semiconductor (CMOS) technology, recent large‐scale neural recording technologies are significantly increasing the number of single cells/units which can be recorded simultaneously at multiple sites of the brain.^[^ ^1^ ^]^ CMOS‐based shank silicon probes cover the important brain regions including the hippocampus, cortex, and thalamus, observed in rats and mice, to provide long‐term stable recording after chronic implant.^[^ ^2^ ^]^ With such CMOS‐based probes, extracellular action potentials from around 1000 individual single neurons could be monitored simultaneously from different brain structures, providing global insight into neural activities to understand neural network function in animal brains.^[^ ^3^ ^]^ Using MEA for in vitro and ex vivo, CMOS‐MEA development has progressed. High‐density (HD) CMOS‐MA capable of simultaneous recording signal from approximately 1000 electrodes to a maximum of 19000 electrodes has been developed.^[^ ^4^ ^]^ Axonal conduction measurements in cultured neurons and electrical activity patterns in brain slices and retinal tissue have been reported.^[^ ^5^ ^]^ The development of field potential imaging by HD‐CMOS‐MEA makes it possible to link the structure and electrical activity of neural circuits at the single‐cell level. The demand for in vitro electrical activity measurement of the nervous system using MEA has increased during the recent years. The discovery of human iPS cells (hiPSC) has made it possible to use human neurons.^[^ ^6^ ^]^ This is because the elucidation of human neurological diseases and the development of new drugs will advance from extrapolability to humans and the use of human neurons derived from disease patients. The toxicity evaluation of pharmaceuticals using healthy hiPSC‐derived neurons and the electrical activity analysis of neurons from diseased patients has been reported.^[^ ^5^, ^7^ ^]^ Additionally, hiPSC‐derived peripheral neurons are used in evaluating pain associated with pain‐related substances and anticancer drug administration compounds.^[^ ^8^ ^]^ Some brain organoids which mimic the 3D structure of humans have been studied with MEA measurement method used as a functional measurement method for brain organoids.^[^ ^9^ ^]^ HD‐CMOS‐MEA captures activity and axonal conduction at the single‐cell level because of its high spatiotemporal resolution. By measuring activity at the single‐cell level, it will be possible to quantify propagation patterns via synapses in the neuronal networks and cerebral organoids using extracellular potentials. This will contribute to in vitro drug efficacy assay and neurological disease research. Peripheral nerves which control pain are known to have different channels expressed in various that have different cells responses to compounds.^[^ ^8^, ^10^ ^]^ Additionally, the peripheral neuropathy by the compound is divided into toxicity to somas, axons, and myelin. By evaluating pain‐related substances using the firing pattern of a single cell, it will become a new pain evaluation system based on the electrical activity. Additionally, when combined with axonal conduction measurements, it will become an assessment method to predict soma, axonal, and myelin toxicity based on electrical activity.

**Figure 1:**
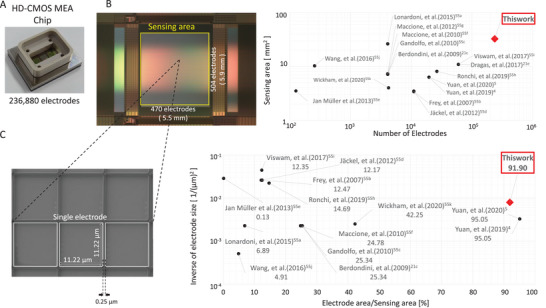
High‐density (HD) CMOS‐MEA with 236880‐electrode and wide measurement area. A) Overview of HD‐CMOS‐MEA chip. B) Sensing area (5.5 mmH × 5.9 mmV). C) Electron microscope image of single electrode size (11.22 µm × 11.22 µm, Separation=0.25 µm). D) Comparison of the number of electrodes to the measurement area.^[^ ^4^, ^5^, ^21^, ^55^ ^]^ E) Comparison of the reciprocal of the electrode size to the number of electrodes per measurement area.^[^ ^4^, ^5^, ^21^, ^55^ ^]^

HD‐CMOS‐MEA is a technology that enables the measurement of nerve activity and axonal conduction at the single‐cell level. However, to analyze the neural network activity state in vitro and ex vivo, it is a challenge to develop a measurement system which combines the following five points: 1) high spatial resolution, 2) high temporal resolution, 3) wide range measurement, 4) high signal to noise ratio (SNR), and 5) real‐time measurement with fast scan speed. Our HD‐CMOS‐MEA features 236 880 platinum (Pt) electrodes with a size 11.22 × 11.22 µm and separation of 0.25 µm, and 33840 readout channels operating at 70 kHz with a noise level of 9.43 ± 1.77 µV rms. In addition, our MEA enables simultaneous measurement of a wide area of 5.51 × 5.91 mm^2^ (**Figure** **1**). This MEA employs a disaggregated differential amplifier and one‐sided feedback and auto‐zero circuit to reduce the pixel size and suppress the noise. Moreover, it integrates single‐slope ADCs, a 4.752 Gbps per ch output interface, and a stacked device structure to enhance the readout speed. Due to densely arranged electrodes, single‐cell signals can be recorded with multiple electrodes. The noise is sufficiently suppressed by processing an average or a correlation between the signals from these electrodes, and small signals, such as axonal signals or action potentials of immature neurons, can be detected. The large sensing area can visualize the entire neuronal network activities while recording signals at a cellular resolution.

Human iPSC‐derived central neurons, which are samples that could be extrapolated to humans, and brain organoids, which are samples that could be extrapolated in vivo, as well as brain slices that have frequently been used as tissues with preserved 3D structures, are effective samples for evaluating the central neurotoxicity of compounds. Additionally, the measurement of sensory neurons is effective in verifying the applicability to peripheral neuropathy assessment. Therefore, in this study, using our HD‐CMOS‐MEA, we demonstrated 1) detection of detailed propagation patterns between regions in brain slices, 2) quantified synaptic strength and drug response based on single‐cell‐level firing patterns in human iPS central neural networks, and 3) detected of responses to pain‐related substances based on the firing patterns of single‐cell units in sensory neurons, and 4) detailed activity measurements and drug responses at the single‐cell level in human cerebral organoids. These results are helpful for 1) understanding circuit operation principles based on propagation patterns obtained by detailed interregional propagation measurements in brain slices; 2) pharmacology based on single‐cell‐level firing patterns and synaptic strengths in the central neuronal network; 3) a novel assessment method for drug‐induced peripheral neuropathy based on single cell‐level firing patterns; and 4) neurological disease research and pharmacological tests based on propagation patterns of human cerebral organoids. These assays will provide a new and effective method for understanding neural circuit activity, including neurological diseases, and evaluating the efficacy and toxicity of pharmaceuticals, based on detailed propagation patterns of nerves in vitro and ex vivo.

## 2 Results

### 2.1 Performance of a 236 880 Electrode CMOS‐MEA and Comparison with Conventional CMOS‐MEAs

Figure 1A–C shows the appearance of HD‐CMOS‐MEA with 236 880 platinum (Pt) electrodes, having a sensing area in 5.5 mmH × 5.9 mmV. It has an electron microscope image of the electrode surface with size 11.22 × 11.22 µm and separation of 0.25 µm. The number of electrodes and sensing area is the largest compared to the conventional CMOS‐MEAs (Figure 1D). The reciprocal value of the electrode area per electrode, which indicates the microscopic of the electrodes, is the fifth overall, but the electrode density to the measurement area is 91.9%. This is an HD‐CMOS‐MEA that has the highest number of electrodes compared to other available MEA. It can measure nerve activity with a high spatial resolution because it uses microelectrodes and covers almost the entire sensing area (Figure 1E).

### 2.2 Validation of Large‐Scale and High Spatiotemporal Resolution Recording Capabilities on Mouse Brain Slice

**Figure 2:**
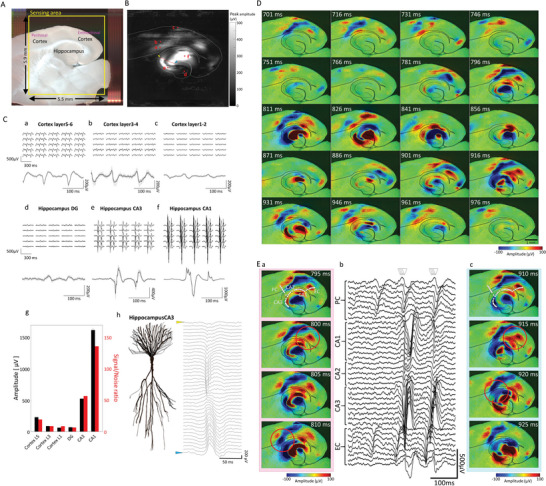
Detailed measurement of spontaneous activity patterns in the hippocampus and cerebral cortex in acute mouse brain slice. A) Mouse brain slice on HD‐CMOS‐MEA. The yellow square indicates a sensing area of 5.5 mm × 5.9 mm. B) Peak amplitude heatmap in 236880 electrodes and schematic diagram of cortical and hippocampal areas. (a) Cortical layer 5. (b) Cortical layer 3. (c) Cortical layer 1. (d) Hippocampus DG. (e) CA3. (f) CA1. C) Raw waveforms in the cortical and hippocampal regions. The waveforms of 25 electrodes in the regions (a) to (f) are indicated by red dots in (B) and the average waveform is a combination of the waveforms of 25 electrodes. (g) Voltage values (black) and signal‐to‐noise ratio (red) of the average waveform. (h) Presumptive schematic of CA3 pyramidal neuron morphology and waveforms acquired at the electrodes indicated via the red line in (B). Waveforms are arranged from yellow to blue arrows in (B). D) Temporal changes of voltage heatmaps in spontaneous activities in the hippocampus and cortex regions. The propagation pattern for 275 ms is shown as a voltage heat map at 15 ms intervals. E) Synchronized firings between hippocampal and cortical regions. Temporal changes of voltage heatmaps for two synchronous firings first on left (a), second on right (c), for 15 ms at 5 ms interval). The raw waveform of the white dotted line part of the heat map (hippocampus CA3, CA2, CA1, perirhinal cortex, entorhinal cortex regions) was picked up (b). Red circles indicate synchronized firing between hippocampal CA1 and EC region, hippocampal CA3 and PC region.

Our HD‐CMOS‐MEA with large‐scale sensing area and high spatial resolution was used in recording extracellular activity in mouse sagittal brain slices. The HD‐CMOS‐MEA has sensing regions which allows simultaneous measurements from the cerebral cortex, hippocampus, midbrain, thalamus, and caudate putamen (**Figure** **2**A), with spontaneous activity observed from the cerebral cortex and hippocampus (*n* = 1 sample, Figure 2B–D). Local field potential (LFP) reveals detailed propagation patterns for several hundred milliseconds in the hippocampus, perirhinal cortex (PC), and entorhinal cortex (EC) regions (Movie S1, Supporting Information, Figure 2D). The PC and EC regions were estimated from images of brain slices. Figure 2C‐a–f shows the waveforms of 25 electrodes measured in 5 vertical and 5 horizontal electrodes (3147.21 µm^2^) in each region, which are indicated by the red dots in Figure 2B, and their average waveforms. The average waveform voltage values for the 25 electrodes were 230.0 ± 19.3 µV in cortical layer 5, 86.6 ± 8.7 µV in cortical layer 3, 62.0 ± 8.5 µV in cortical layer 1, 85.0 ± 9.2 µV in the entorhinal cortex, 68.8 ± 6.6 µV in the dentate gyrus, 525.3 ± 56.6 µV in CA3, and 1622.1 ± 136.2 µV in CA1. The signal‐to‐noise ratio (SNR) was 19.3 in cortical layer 5, 8.7 in cortical layer 3, 8.5 in cortical layer 1, 6.6 in the dentate gyrus (DG), 56.7 in CA3, and 136.2 in CA1 (Figure 2C‐g). The intensity of the signal depends on 1) the number of neurons firing synchronously via synaptic propagation and 2) adhesion between the electrode and tissue. Therefore, it is thought that the hippocampus has a larger number of neurons that maintain and synchronize the circuit structure in terms of slice preparation and that the signal intensity tends to be stronger than that of the cerebral cortex. Although the signal intensity differed depending on the brain region, it was confirmed that the activity could be acquired with a high SNR from the spontaneously active region.

Surprisingly, due to the high spatiotemporal resolution, the anatomical structures of the hippocampus and cerebral cortex are highlighted from the potential waveforms by positive and negative potential imaging (Figure 2D, Movie S1, Supporting Information). Additionally, we can find the relationship between pyramidal neuronal morphology in CA3 and sink and source in LFP at a certain time. From the radiatum layer to the lacunosum‐molecular layer, the potential changed from negative (sink) to positive (source) (Figure 2C‐h). Local propagation within layer 5 of the cortex, propagation from the EC to the PC, propagation from the hippocampal DG to CA3 and CA3 to CA1, and input from CA3 to CA1 via Schaffer collaterals were observed along the brain regions. (Figure 2D, Movie S1, Supporting Information).

We have used low Mg^2+^ 0.1 × 10^‐3^ m to induce epileptiform activity in vitro which resembles Inter‐Ictal events (I‐IC, Movie S1, Supporting Information, Figure 2D,E) recorded in humans with EEG before or after an epileptic seizure.^[^ ^11^ ^]^ Figure 2D shows that the cortical region fires independently at around 700 ms, while synchronous firing in the cortical and hippocampal regions was confirmed at about 800 and 900 ms. In both synchronous firings (Figure 2E‐b), we observed a phenomenon in which the firing from the EC was linked at the CA1 and CA3 sites. As shown by the circles in Figure 2Ea,c, we observed a phenomenon in which the firing from the EC to CA1 sites and from the PC to CA3 sites were synchronous firings. The perirhinal cortex (PC) and entorhinal cortex (EC) regions indicated via white dotted lines were estimated from images of brain slices. The occurrence of the event in each region for 8 s was observed 7 times in CA3, 10 times in CA2, 13 times in CA1, 7 times in PC, and 12 times in the EC. CA3 propagated through the 0.25 ± 0.03 mm^2^ at a velocity of 8.81 ± 3.93 mm^2^ s^‐1^, CA2 propagated through the 0.11 ± 0.02 mm^2^ at a velocity of 2.55 ± 0.59 mm^2^ s^‐1^, CA1 propagated through the 0.13 ± 0.02 mm^2^ at a velocity of 4.00 ± 0.98 mm^2^ s^‐1^, PC propagated through the 0.25 ± 0.03 mm^2^ at a velocity of 3.04 ± 0.51 mm^2^ s^‐1^, and EC propagated through the 0.22 ± 0.03 mm^2^ at a velocity of 6.07 ± 1.28 mm^2^ s^‐1^. The projection pathways between EC and hippocampal regions include the corticoammonic nucleus pathway with direct input from the EC to the CA1 area, and the trisynaptic pathway via the EC, dentate gyrus, CA3, and CA1 area.^[^ ^12^ ^]^ Voltage heatmap data was electrical activity consistent with these projection pathways.

### 2.3 Propagation of Gamma Waves and Sharp‐Wave Ripple (SPW‐R) Waves in Brain Slice

**Figure 3:**
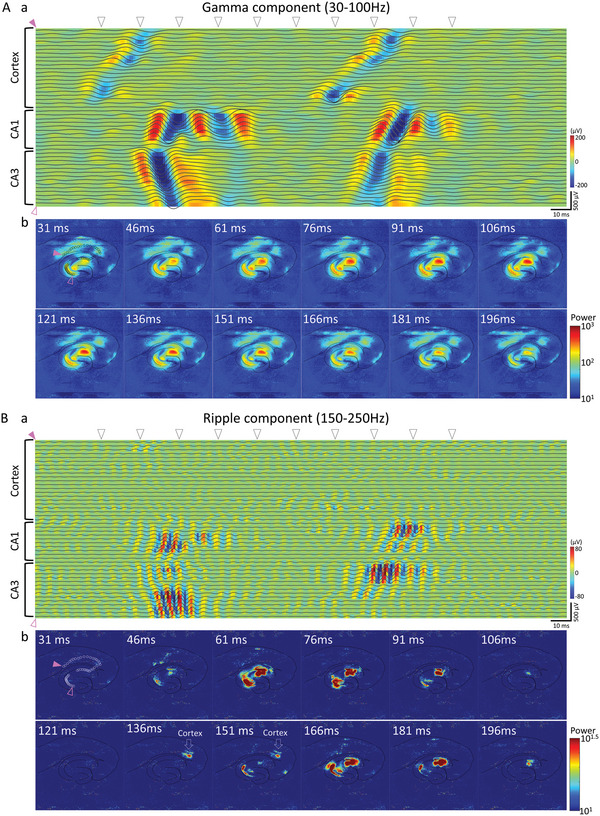
Propagation of Gamma and Ripple waves in hippocampus and cortex. A‐a) Waveforms and voltage heatmaps for 250 ms obtained by bandpass filtering (30–100 Hz) of the signals in the cortical area, CA1, and CA3 areas. (b) Time‐lapse of the intensity heatmap (scalogram) of the gamma waveband obtained by wavelet transform. The waveform and voltage heatmaps picked up in (a) the data acquired at the electrode indicated by the black dot in 31 ms image. Pink triangles indicate the starting and ending electrodes. B‐a) Waveforms and voltage heatmaps for 250 ms obtained by bandpass filtering (150–250 Hz) the signals in the cortical area, CA1, and CA3 areas. (b) Time‐lapse of scalogram of the ripple waveband. Arrows at 136 ms and 151 ms indicate that ripple components were observed in the cortex area. The triangle arrows in the top of the waveforms and voltage heatmaps (a) indicate times of time‐lapse images. The time of the triangle arrows was the same as the triangle arrows in Figure 2E.

The frequency characteristics of LFP have been extensively studied in the hippocampus and cerebral cortex, and are an effective index for evaluating brain function. The gamma frequency has been implicated in cognitive function, and sharp wave‐ripple (SPW‐R) waves are a marker of epilepsy.^[^ ^13^ ^]^ Gamma and SPW‐R waves have been reported to have stronger connections with the cortex.^[^ ^14^ ^]^ Here, we investigated the spatiotemporal distribution of gamma and ripple wave components in the hippocampus and cortex (**Figure** **3**). Gamma wave propagation was observed within the cortex and the hippocampal CA1 and CA3 regions. In the hippocampus, a pattern of propagation from CA1 to CA3 and from CA3 to CA1 was observed (Figure 3A‐a). Like gamma waves in the hippocampus, ripple waves propagated between CA3 and CA1 (Figure 3B‐a,b). Few ripple waves were observed in the cortex, but interestingly, local oscillations were observed in the cortex on the CA1 side. These results indicate a connection between the hippocampus and cerebral cortex of ripple waves. Our brain slice measurement using HD‐CMOS‐MEA could investigate the propagation between brain regions according to frequency.

### 2.4 Network Analysis and Single Neuron Analysis in Drug Response of Human iPSC‐Derived Cortical Neurons

**Figure 4:**
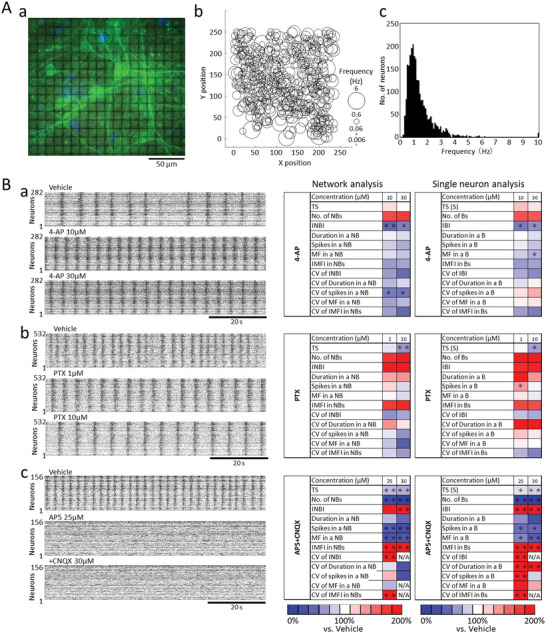
Network analysis and single neuron analysis to compounds responses in hiPSC‐derived cortical neurons. A) Spontaneous activity frequency of human iPS cell‐derived cortical neurons per neuron. Spontaneous activity measurement and pharmacological tests were carried out after 42 days of culture. (a) Immunostaining image of human iPS cell‐derived neurons cultured on CMOS‐MEA (18 × 18 = 324 electrodes) at six weeks of culture. Green: *β*‐tubulin III, Blue: Hoechest33258. (b) Spontaneous activity frequency per neuron detected by 235 × 252 = 59220 electrodes. The size of the circle indicates the spontaneous firing frequency of 532 neurons. (c) Histogram of spontaneous activity frequency per neuron (n = 9 CMOS‐MEAs, *n* = 2569 neurons). B) Representative raster plots and heatmaps of network analysis and single neuron analysis for drug administration. (a) Vehicle, 4‐AP 10 × 10^‐6^ m, 30 × 10^‐6^ m. (b) Vehicle, PTX 1 × 10^‐6^ m, 10 × 10^‐6^ m. (c) Vehicle, AP‐5 25 × 10^‐6^ m AP‐5+CNQX 30 × 10^‐6^ m. Heat map shows % versus vehicle = 100% (*n* = 3, *: *p* ≦ 0.05, **: *p* ≦ 0.01 versus vehicle, Dunnett's test). N/A: unable to calculate due to loss of burst. Parameters in network analysis [TS: Total spikes, No. of NBs: number of network bursts, INBI: Inter NB Interval, Duration in a NB, Spikes in a NB, MF in a NB: Maximum frequency in a NB, IMFI in NBs: Inter MF Interval in NBs, CV of INBI: Coefficient of variance (CV) of Inter NB Interval, CV of Duration in a NB, CV of spikes in a NB, CV of MF in a NB, CV of IMFI in NBs]. Parameters in single neuron analysis [TS(S): Total spikes per single neuron, No. of Bs: number of bursts, IBI: Inter burst Interval, Duration in a B, Spikes in a B, MF in a B: Maximum frequency in a B, IMFI in Bs: Inter MF Interval in Bs, CV of IBI: Coefficient of variance (CV) of Inter B Interval, CV of Duration in a B, CV of spikes in a B, CV of MF in a B, CV of IMFI in Bs.]

We have evaluated drug responsiveness in hiPSC‐derived cortical networks. **Figure** **4**A‐a shows the morphology of hiPSC‐derived neurons cultured on 324 electrodes (18 × 18 electrodes). We observed that the soma of a single neuron spanned approximately four electrodes. To detect single‐cell firing, electrodes with identical firing patterns were combined. Specifically, the firing frequency histogram of 1 ms bins for each electrode was calculated, and electrodes with a cosine similarity of ≥0.3 in histograms between adjacent electrodes were combined as electrodes of the same neuron. The locations and firing frequencies of each neuron are shown in Figure 4A‐b. The center of the circle represents the centroid position of each neuron, and the size of the circle depicts the firing frequency of each neuron. The cell images shown in Figure 4A‐b are different from the electrodes shown in the fluorescence photograph of Figure 4A‐a As a result of spontaneous activity measurement for 1 min, firing was detected at an average of 285 ± 52 neurons per well (*n* = 9 wells, Movie S2, Supporting Information). Figure 4A‐c shows the distribution of firing frequency in all neurons (*n* = 9 wells). The firing frequency ranged from 0.029 to 9.994 Hz, and the mode was 0.9 Hz. The time course of the average firing frequency is shown in Figure S1 (Supporting Information). These results indicate that HD‐CMOS‐MEA system can analyze the spontaneous activity of the hiPSC‐derived cortical network on a cell‐by‐cell basis. Figure 4B shows a raster plot for each neuron, a heat map of burst parameters in the network, and a heat map of burst parameters in single neurons, when 4‐AP, PTX, and AP5 + CNQX were administered. Each analysis parameter was calculated and normalized with the vehicle data of each well as 100%. The data before normalization are listed in Table S1 (Supporting Information). Black bands in Figure 4B indicate network bursts (NB), and NB was observed in all wells before drug administration. 4‐AP administration increased the total spikes (TS) and total spikes per single neuron [TS(s)], number of NBs, and burst in a single neuron (No. of Bs), and decreased the CV of spikes in an NB and Max Frequency in a B (MF) (Figure 4B‐a). Max frequency was calculated from the maximum value of the histogram of the number of firings during one burst. The administration of picrotoxin (PTX), a GABA‐A receptor blocker, increased the duration in NB and B, the spikes in B at 1 × 10^‐6^ m (Figure 4B‐b). It was confirmed that GABA‐A receptors are functionally expressed in hiPSC‐derived neural networks. These results also indicate that both the network and single neuron analyses can detect the response of the seizurogenic compounds.

AP5 (25 × 10^‐6^ m), an antagonist of NMDA‐type glutamate receptors, decreased the 5 parameters (TS and TS(S), No. of NBs and No. of Bs, duration in a NB and B, Spikes in a NB and B, MF in a NB and B), and increased 3 parameters [Inter MF Interval (IMFI) in NB and B, CV of Inter NB and B Interval, CV of IMFI in NB and B]. CV of duration in a B and CV of spikes in a B changed only in single neuron analysis. Additional administration of CNQX 30 µM, an AMPA receptor inhibitor, also decreased the 5 parameters and increased 2 parameters (Inter NB and B interval, IMFI in NB and B). CV of INBI and IBI, CV of MF in a NB, CV of IMFI in a NM and B were not calculated because CNQX reduced or eliminated NB and B (Figure 4B‐c). From these results, it was confirmed that the function of both NMDA and AMPA‐type glutamate receptors in cultured hiPSC‐derived cortical neurons.

Interestingly, the coefficient variance (CV) of duration in an NB and B, CV of spikes in an NB and B, and CV of MF in an NB and B were different between network analysis and single neuron analysis of four compounds. These results indicate that adding single neuron analysis parameters increases the amount of information and provides new information in evaluating the drug responsiveness of neural networks.

### 2.5 Evaluation of Synaptic Strength by Compound Administration

**Figure 5:**
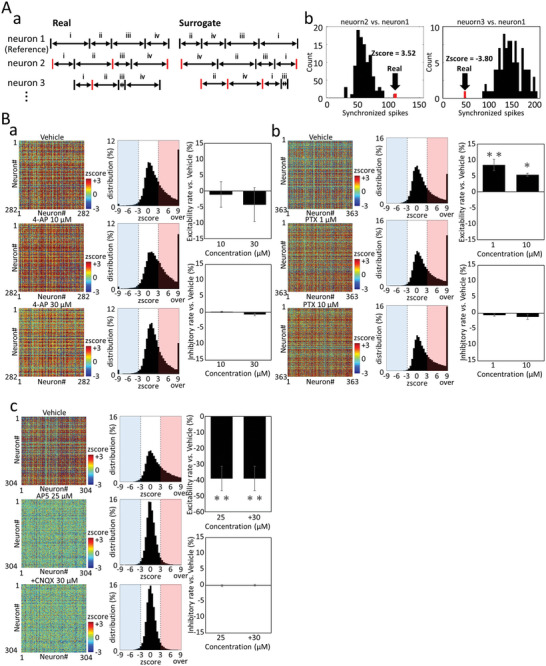
Changes in the synaptic strength due to compound administration in hiPSC‐derived cortical neurons. A) Z score calculation to quantify connection strength between neurons. (a) Raster plot of three representative neurons. Left: real data, right: random surrogate data. Red raster plots show spikes that fire within 100 ms of neuron1 firing. (b) Synchronized spike histogram calculated from spike trains of neuron2 and neuron1 (left) and synchronized spike histogram calculated from spike trains of neuron3 and neuron1 (right). Black indicates random surrogate data, red indicates a synchronized spike of real data. B) Changes in Z score due to compound administration. (a) vehicle (Top), 4‐AP 10 × 10^‐6^ m (middle), 4‐AP 100 × 10^‐6^ m (Low). (b) vehicle (Top), PTX 1 × 10^‐6^ m (middle), PTX 10 × 10^‐6^ m (low). (c) Vehicle (Top), AP‐5 25 × 10^‐6^ m (middle), AP‐5 25 × 10^‐6^ m+CNQX 30 × 10^‐6^ m (Low). Left: Matrix of Z scores between each neuron before and after compound administration. The color scale is −3 ≤ Z score ≤ 3. Middle: Z score histogram of all connections. The red area (Z score ≥ 3) indicates an excitable connection, and the blue area (Z score ≤ 3) indicates an inhibitory connection. Right: Excitability connections percentage change (top, Ave. ±S.E., *n* = 3) and inhibitory connections percentage change (low, Ave. ±S.E., *n* = 3) due to compounds administration (* : *p* ≦ 0.05, ** : *p* ≦ 0.01 vs vehicle, Dunnett's test).

To investigate changes in synaptic strength due to the compound administration, firing time‐series data of each neuron was extracted, and the number of spikes fired within 100 ms for each spike of each neuron (synchronized spike) was counted for all combinations of neurons. Next, 100 surrogate time‐series data were created by randomly rearranging the interspike intervals (ISI) calculated from the time‐series data of each neuron, and synchronized spikes were counted. **Figure** **5**A‐a shows synchronized spikes (red bar) fired within 100 ms with Neuron1 as a reference for real and surrogate time‐series data. The Z score for the number of synchronous spikes was calculated from the real synchronized spikes and 100 surrogate synchronized spikes. Figure 5A‐b shows histograms of synchronized spikes from the combination of Neuron1 and Neuron2, and Neuron1 and Neuron3. The black bar represents 100 surrogate data, and the red represents the real data. The Z score for Neuron1 versus Neuron2 was 3.52, and for Neurons1 versus Neuron3 was −3.80. When the *Z* score of the real synchronized spike exceeded 3, the combination of neurons was recognized as an excitable connection. Conversely, when the Z score was less than −3, it was recognized as an inhibitory connection. The color map on the left in Figure 5B‐a–c shows the Z score matrix between each neuron, and the middle shows the Z score histogram for all connections. The red area of the histogram indicates an excitable connection, and the blue area indicates an inhibitory connection. Before compound administration, the number of combinations with a Z score of ≧3 was 33.5% ± 4.3% (*n* = 9 wells), which indicates the percentage of excitatory connections that were formed. For 4‐AP, the percentage of excitability connection tended to decrease, −1.08% ± 4.02.% at 10 × 10^‐6^ m and −4.25% ± 5.30% at 30 × 10^‐6^ m, and the inhibitory connections changed slightly, 0.176% ± 0.20% at 10 × 10^‐6^ m, −0.66% ± 0.39% at 30 × 10^‐6^ m (Figure 5B‐a). On the other hand, PTX increased the percentage of excitability connections, 8.44% ± 1.80% (*p* < 0.01) at 1 × 10^‐6^ m and 5.38 ± 0.47% (*p* < 0.05) at 10 × 10^‐6^ m (Figure 5B‐b). The inhibitory connections changed slightly, −0.63% ± 0.35% at 1 × 10^‐6^ m and −1.17% ± 0.76% at 10 × 10^‐6^ m (Figure 5B‐b). These results indicate that PTX enhances excitatory coupling and shifts to a regular firing pattern compared to 4‐AP. Administration of 25 × 10^‐6^ m AP5 decreased the percentage of excitability connections to −39.04% ± 7.68%, and the subsequent addition of 30 × 10^‐6^ m CNQX did not change the percentage, which was −39.06% ± 7.62% (Figure 5C‐c). Inhibitory connection was unchanged, −0.08% ± 0.27% at AP5 25 × 10^‐6^ m and 0.07% ± 0.27% at +CNQX 30 × 10^‐6^ m. This indicates that excitatory synaptic connections were blocked, which results in random firing.

**Figure 6:**
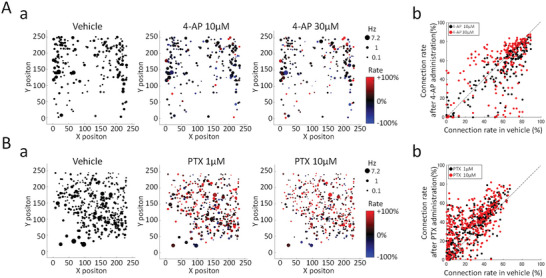
Changes in firing frequency of single neuron and excitability connection rate (ECR) after 4‐AP and PTX administration. There were positions of neurons where the firing was detected on the 59220 electrodes (235 × 252 electrodes) and firing frequencies in single neurons. The firing frequency is indicated by the size of the circle. The size of the circle indicates the firing frequency. The ECR value of each neuron in vehicle is defined as 100%, and the ECR increase/decrease due to drug administration is shown in color. A‐a) Vehicle, 4‐AP 10 × 10^‐6^ m, 30 × 10^‐6^ m. B‐a) Vehicle, PTX 1 × 10^‐6^ m, 10 × 10^‐6^ m. A‐b) 4‐AP 10 × 10^‐6^ m, 30 × 10^‐6^ m. B‐b) Vehicle, PTX 1 × 10^‐6^ m, 10 × 10^‐6^ m.

Among the difference in reactivity between 4‐AP and PTX, we calculated the changes in the firing frequency of one neuron and the proportion of connections (with a Z score of 3 or more) between one neuron and other neurons (excitability connection rate: ECR, ECR = number of Z score ≧3/number of neurons‐1*100%) (**Figure** **6**). Figure 6A‐a shows the distribution of the firing frequency of single cells before and after administration of 4‐AP (*n* = 193 neurons, maximum firing frequency: 6.47 Hz) and changes in ECR. The size of the dots indicates the firing frequency, and the color indicates the increase/decrease of the ECR for the vehicle (100%). At 10 × 10^‐6^ m of 4‐AP, the percentage of neurons with increased ECR was 22.8% and the percentage of decrease was 75.6%, and at 30 × 10^‐6^ m, the percentage of neurons with increased ECR was 50.8% and the percentage of decrease was 47.7%. The rate of unchanged ECR was 1.6% for both 10 × 10^‐6^ m and 30 × 10^‐6^ m. At low concentrations, the ECR of about 80% of the cells decreased; at high concentrations, the rate of increase and decrease was approximately similar. Looking at the size of the circles in Figure 6A‐a, there was no significant finding in firing frequency of each neuron after 4‐AP administration. Figure 6A‐b shows distribution maps of connection rates after 4‐AP administration. The vertical axis represents the ECR after administration, the horizontal axis represents the ECR during the vehicle, and the dotted line represents the line with no change. 4‐AP 10 × 10^‐6^ m showed a tendency to reduce neurons with a vehicle ECR of 50% or more, and 4‐AP 30 × 10^‐6^ m showed a tendency for increasing neurons and decreasing neurons sparsely distributed regardless of the vehicle ECR. Figure 6B‐b shows the distribution of single‐cell firing frequency (*n* = 363 neurons, maximum firing frequency: 7.24 Hz) and changes in ECR before and after PTX administration. At 1 × 10^‐6^ m PTX, the percentage of neurons with increased ECR was 76.9%, the percentage of decrease was 22.3%, and the percentage of neurons without change was 0.8%. At 10 × 10^‐6^ m, the percentage of neurons with increased ECR was 76.9%, the percentage of decreased was 22.0%, and the percentage of neurons without change was 1.1%. PTX increased the ECR about 80% of the neurons from low concentrations, and the tendency was almost the same even at high concentrations. Additionally, changes in the firing frequency of a single neuron were observed to change compared to 4‐AP (Figure 6B‐a). Figure 6B‐b shows the distribution map of the connection rate after PTX administration. At both PTX 1 × 10^‐6^ m and PTX 10 × 10^‐6^ m, many neurons showed an increase in ECR, and the range of increase in neurons with a vehicle ECR of 20% or less was extensive. PTX 10 × 10^‐6^ m tended to have a more extensive ECR fluctuation range than 1 × 10^‐6^ m. Compared to 4‐AP, PTX increased the number of neurons with increased ECR with change in firing frequency of single neurons.

These results showed that the analysis of the intercellular Z score calculated from the firing timing of single neuron is an analysis method for changes in the synaptic strength.

### 2.6 Spontaneous Activity Pattern of DRG Neurons on a Cell‐by‐Cell Basis

**Figure 7:**
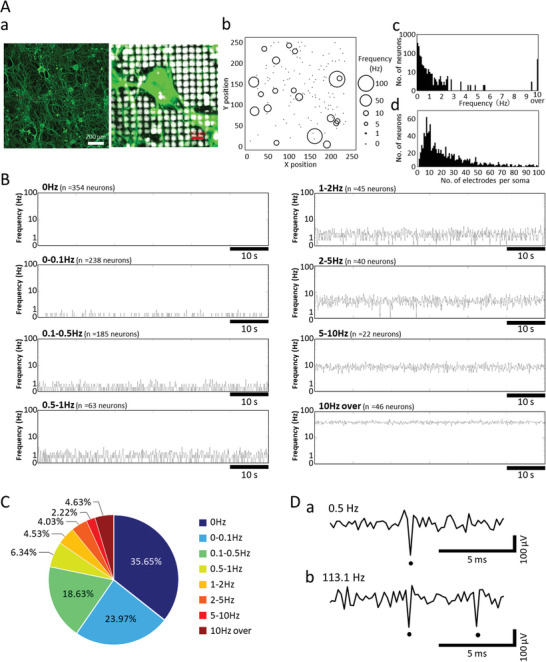
Spontaneous activity frequency distribution in cultured DRG neurons. A) Immunostaining image of DRG neurons (*β*‐tubuline III) at 6 WIV (weeks in vitro) cultured on HD‐CMOS‐MEA (a). Enlarged view near the soma (b). DRG neurons detected on the 235 (X‐axis) × 252 (Y‐axis) = 59220 electrodes and spontaneous activity frequency. The size of the circle indicates the frequency of spontaneous activity from 0 Hz to 100 Hz (c). Histogram of spontaneous activity frequencies of DRG neurons (bin = 100 ms, *n* = 993 neurons, *n* = 7 HD‐CMOS‐MEAs) (d). Histogram of number of electrodes per soma calculated from spontaneous activity of DRG neurons (bin = 1 electrodes, *n* = 993 neurons, n = 7 HD‐CMOS‐MEAs). B) The spontaneous activity of single DRG neurons is a typical firing pattern per minute in each frequency band. C) Percentage of DRG neurons in each spontaneous firing frequency band. D) Comparison of voltage waveforms of DRG neurons that exhibited low‐frequency firing (a) and exhibited firing rates of >100 Hz (b). Dots show spikes.

**Figure 8:**
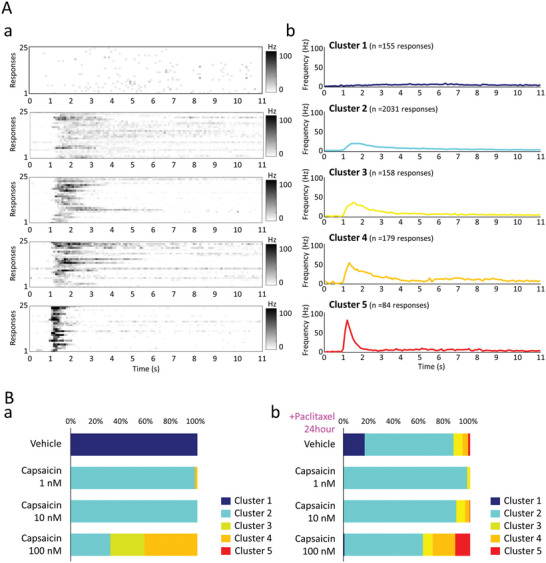
Responses to capsaicin in the presence and absence of paclitaxel. A) Clustering of evoked responses in capsaicin administration. Induced response of 25 typical neurons in each of the 5 clusters (bin = 100 ms). The firing frequency of 0 to 100 Hz is shown in gray scale (a). Time course of average firing frequency for each of the 5 clusters (b). B) Cluster ratio of the evoked response to capsaicin 1 × 10^‐9^, 10 × 10^‐9^, 100 × 10^‐9^ m administration (a). Cluster ratio of evoked response to capsaicin 1 × 10^‐9^, 10 × 10^‐9^, 100 × 10^‐9^ m administration in the presence of paclitaxel 10 × 10^‐9^ m for 24 h (b).

We have evaluated the detection of cell‐by‐cell spontaneous activity patterns of DRG neurons and their responsiveness to pain‐related compounds. **Figure** **7**A‐a shows DRG neurons cultured on HD‐CMOS‐MEA at 6 WIV (weeks in vitro). A peculiar appearance of DRG neurons with different soma sizes was observed on HD‐CMOS‐MEA. The soma of about 100 µm in the major axis direction was located on 29 electrodes (Figure 7A‐a). The spontaneous activity of each neuron was measured for 60 s, and the frequency of each neuron with spontaneous activity was detected (*n* = 993 neurons, *n* = 7 wells). To detect the soma of DRG neurons, the average cosine similarity between the waveforms of the surrounding eight electrodes and waveform of the center electrode was calculated for all electrodes except the electrodes located at the ends of the four sides. Since the action potential of one neuron is detected using multiple electrodes, the cosine similarity tends to be high. Figure 7A‐b shows the soma position and firing frequency detected on one HD‐CMOS‐MEA in the size of a circle. Identification of soma location included neurons that responded to capsaicin (**Figure** **8**). Therefore, neurons that were not spontaneously active were identified at the time of spontaneous activity measurement. Figure 7A‐c shows the distribution of spontaneous activity frequencies of all 993 neurons. Unlike the central nervous system, DRG neurons have no synchronous activity and are spontaneous activities without synaptic transmission.^[^ ^8a^ ^]^ Figure 7A‐d shows the distribution of electrode numbers per soma of all 993 neurons. The average value and standard deviation of electrode numbers per soma were 23.68 ± 20.98 electrodes, and the mode was 7 electrodes.

Figure 7B shows a 1 min firing histogram of typical neurons firing in each frequency band. Figure 7C shows the percentage of DRG neurons in each spontaneous firing frequency band. The proportion of nonspontaneous neurons is highest at 35.65% (*n* = 354 neurons), 0 < firing frequency (*F*) < 0.1 Hz neurons 23.97% (*n* = 238 neurons), 0.1 ≤ *F* < 0.5 Hz neurons 18.63% (*n* = 185 neurons), 0.5 ≤ *F* < 1 Hz neurons were 6.34% (*n* = 63 neurons), 1 Hz ≤ *F* < 2 Hz neurons were 4.53% (*n* = 452 neurons), 2 Hz ≤ *F* < 5 Hz neurons were 4.03% (*n* = 40 neurons), and 5 Hz ≤ *F* < 10 Hz neurons were 2.22% (*n* = 22 neurons). Frequently spontaneously active neurons above 10 Hz were present at a rate of 4.63% (*n* = 46 neurons). The maximum firing frequency was 113.1 Hz. We found that there are DRG neurons, which exhibit high‐frequency firing above 100 Hz. Figure 7D shows the comparison of voltage waveforms of DRG neurons that exhibited firing rates of >100 Hz and exhibited low‐frequency firing. The waveforms of neurons with high firing frequency were similar to those of neurons with low firing frequency. Therefore, it was confirmed that it was not noise. Based on these results, it was found that DRG neurons have spontaneous activity with different firing frequencies.

### 2.7 Response to Capsaicin in DRG Neurons and Enhancing Effect by Paclitaxel

Next, the evoked response by the administration of capsaicin, an agonist of the TRPV1 channel, was examined. Figure 8A shows the results of clustering the evoked response patterns after capsaicin administration (*n* = 2607 responses, *n* = 653 neurons, n = 5 CMOS‐MEAs). In each cluster, the evoked response of 25 typical neurons is shown (Figure 8A‐a), and the histogram of the average evoked response is shown in Figure 8A‐b. Cluster1 is a sample group that does not show a rapid increase in the number of firings after administration. Cluster2 had a peak frequency of 20.7 Hz after 1.5 s and an average frequency of 7.7 Hz for 10 s. Cluster3 had a peak frequency of 35.8 Hz after 1.5 s and an average frequency of 9.4 Hz for 10 s. Cluster4 had a peak frequency of 52.8 Hz after 1.3 s and an average frequency of 13.2 Hz for 10 s. Cluster 5 showed a strong peak frequency of 80.2 Hz after 1.2 s, after which the firings decreased sharply, with an average frequency of 8.2 Hz for 10 s. These results indicate that each DRG neuron has a different evoked response to capsaicin. Figure 8B‐a shows the concentration‐dependent response of capsaicin. With Capsaicin 1 × 10^‐9^ m administration, Cluster 2 was 97.87%, and with 10 × 10^‐9^ m it was 100%, showing the same response. On the other hand, when 100 × 10^‐9^ m was administered, highly reactive cluster 3 accounted for 27.08% and cluster 4 accounted for 41.67%. Figure 8B‐b shows the capsaicin response results after exposure to the anticancer drug paclitaxel 3 × 10^‐9^ m for 24 h. In the vehicle, cluster 2 was 70.10%, cluster 3 was 7.31%, cluster 4 was 4.32%, and cluster 5 was 1.66%. In capsaicin 1 × 10^‐9^ m, cluster 2 was 97.36%, cluster 3 was 1.98%, cluster 4 was 0.50%, and cluster 5 was 0.00%. In capsaicin 10 × 10^‐9^ m, cluster 2 was 88.76%, cluster 3 was 6.94%, cluster 4 was 3.64%, and cluster 5 was 0.50%. With capsaicin 100 × 10^‐9^ m, cluster 2 was 61.86%, cluster 3 was 7.79%, cluster 4 was 17.74%, and cluster 5, which showed the strongest response at 11.77%, and activation of the TRPV1 channel by paclitaxel administration. The analysis results of the evoked response on a cell‐by‐cell basis suggested that not all cells showed a uniformly strong response, and that the ratio of evoked response characteristics was related to pain intensity. It was also shown that the effect of anticancer drugs could be evaluated from the evoked response pattern of each neuron.

### 2.8 Back Propagation and Forward Propagation in Axon

**Figure 9:**
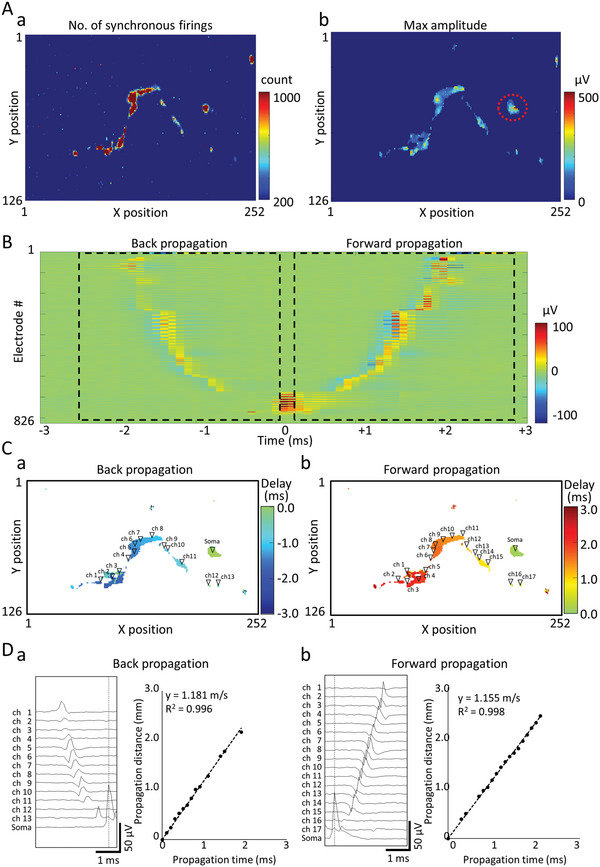
Back propagation and forward propagation in single axonal conduction. A) Identification of soma electrode locations, (a) Number of synchronous firings map, (b) Maximum amplitude map of electrodes with more than 200 synchronous firings detected. The dotted line indicates the soma electrode area. B) Average potential heat map for 3 ms before and after soma firing. C) Conduction path and delay time map, (a) Heat map of back conduction 3 ms before soma firing, (b) Heat map of forward conduction 3 ms after soma firing. D) Electrode voltage waveform used for conduction velocity calculation (left). The dotted line indicates the firing time of the soma. Plot of conduction time and cumulative conduction distance (right). The dotted line is the approximate straight line calculated by the least squares method. (a) Back conduction, (b) Forward conduction.

To identify the conduction path of neurons, the maximum amplitude of each electrode was detected every 0.05 s. Then, synchronous firings were defined as maximum amplitudes with a time delay of less than 1 ms for 4 or more among the 8 electrodes. We counted the synchronous firings of all electrodes from 76.8 s of data, 826 electrodes with more than 200 synchronous firings were derived for analysis (**Figure** **9**A‐a). Next, we created a maximum voltage amplitude map for 76.8 s of 826 electrodes and the dotted line indicates the soma electrode area. (Figure 9A‐b). 100 electrodes detected soma firing, and the maximum amplitude was 521.2 µV. Spike detection of the electrodes (100 electrodes) where the soma is located was performed using a voltage threshold of 200 µV, and 1167 firings were detected. We found a neuron with high‐frequency spontaneous firing at 15.2 Hz. By using the firing time of the soma as a reference, voltage values were extracted 3 ms before and after firing, and the average voltage map of 1167 times was calculated (Figure 9B). Forward propagation was observed for 3 ms after soma firing. Propagation was also observed for about 2 ms before firing the soma. The forward propagation and back propagation had the same route (Movie S3, Supporting Information). Figure 9C shows the conduction pathways around the time of soma firing as a time heat map was shown that the paths of back propagation and forward propagation are the same. Backpropagation was observed 486 times out of 1167 firings, with a probability of 41.6%. Starting from the position of the center of gravity of the soma, the farthest electrode at which firing was obtained every 0.1 ms was selected, and the cumulative linear distance between the electrodes was calculated as the conduction distance. The figure shows the conduction waveforms and conduction velocities of backpropagation and forward propagation. In the waveform, the negative potential (sink) appeared first, followed by the positive potential (source) (Figure 9B,D). The back propagation distance was 2.18 mm, the conduction time was 1.9 ms, and the conduction velocity was 1.181 m s^‐1^ (*R* ^2^ = 0.996). Forward propagation distance was 2.44 mm, conduction time was 2.1 ms, and conduction velocity was 1.155 m s^‐1^ (*R* ^2^ = 0.998). Back propagation and forward propagation had almost the same speed.

### 2.9 Change of Axonal Conduction Velocity to Anticancer Drug Vincristine

**Figure 10:**
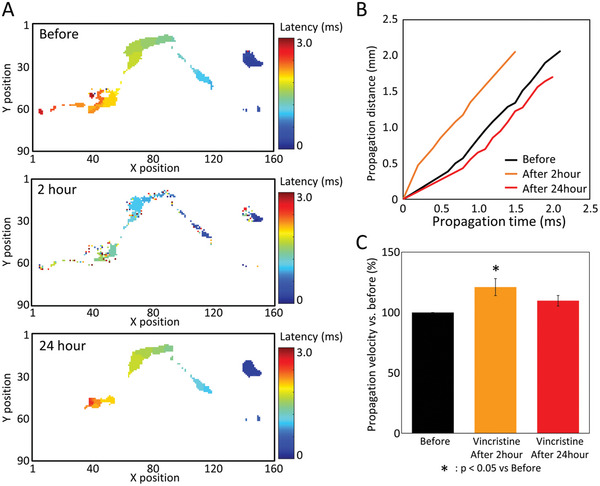
Change of axonal conduction velocity to anticancer drug vincristine. A) Heat map of forward propagation 3 ms after soma firing before and after vincristine administration. The upper row is before administration, the middle row is 2 h after administration, and the lower row is 24 h after administration. B) Plot of conduction time and cumulative conduction distance before and after vincristine administration. C) Change in the conduction velocity by vincristine. The parameters were depicted as the average % change of before (before set to 100%) ± SEM from *n* = 4 wells. Data were analyzed using one‐way ANOVA followed by posthoc Dunnett's test (*p < 0.05 vs before).

Next, we examined how the axonal forward propagation velocity changes with the administration of an anticancer drug, vincristine. **Figure** **10**a shows typical forward propagation patterns before and after vincristine 3 × 10^‐9^ m administration. The number of electrodes detect forward propagation was 719 electrodes before, 436 electrodes 2 h after administration, and 504 electrodes 24 h after administration. A graph of conduction distance versus conduction time for this axon is presented in Figure 10b. The conduction velocity was 1.16 m s^‐1^ (2.44 mm/2.1 ms) before administration, 1.37 m s^‐1^ (2.05 mm / 1.5 ms) after 2 h, and 0.85 m s^‐1^ (1.70 mm / 2.0 ms) after 24 h. Figure 10c shows changes in axonal conduction velocities acquired with separate CMOS‐MEAs (*n* = 4 axons, *n* = 4 wells). Conduction velocity increased by 121.0% ± 7.1% (*p* = 0.0234) 2 h after administration of vincristine and by 109.7% ± 4.4% (*p* = 0.3066) 24 h after administration of vincristine. Two hours after the administration of vincristine 3 × 10^‐9^ m, axonal conduction velocity increased. Data were analyzed using one‐way ANOVA followed by posthoc Dunnett's test.

### 2.10 Detection of Spontaneous Firing Patterns and Drug Responses in Patient‐Derived Cerebral Organoids

**Figure 11:**
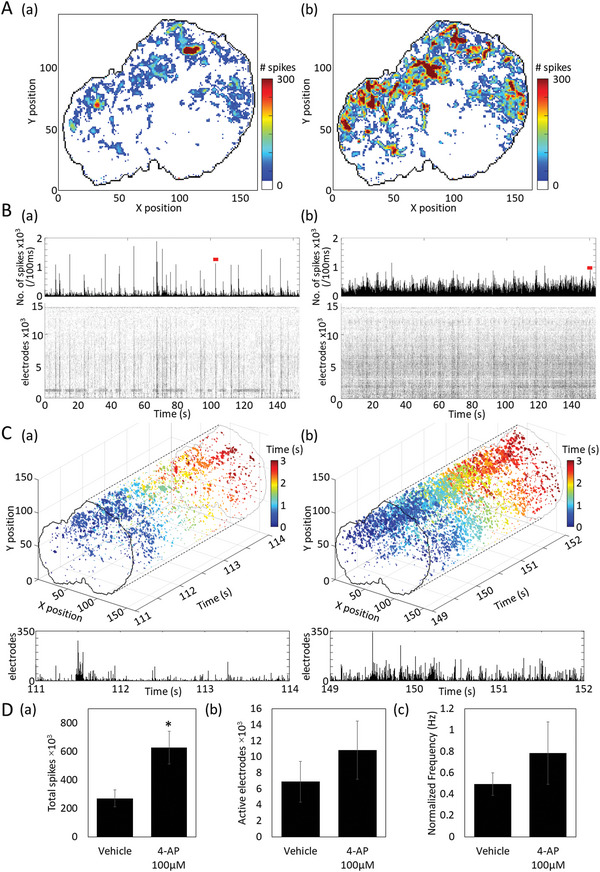
Spontaneous activity measurement and drug response in human cerebral organoids. A) The adhesion surface between human cerebral organoids derived from Rett syndrome patients and CMOS‐MEA is drawn with a black line. A heat map shows the number of firings detected in the spontaneous activity measurement for 153.6 s. (a) Vehicle, (b) 4‐AP 100 × 10^‐6^ m. B) Raster plot of 14612 electrodes on the contact surface and histogram of the number of firings per 100 ms, (a) Vehicle, (b) 4‐AP 100 × 10^‐6^ m. C) Top row: 3 s 3D raster plot of the network burst indicated by the red line in B. The 3D raster plots represent time on the x‐axis and electrodes to which the organoids are in contact in the Y–Z plane. The color scale of the plot indicates time from burst initiation. Bottom: Histogram of the number of firing electrodes per 5 ms for 3 s. (a) Vehicle, (b) 4‐AP 100 × 10^‐6^ m. D) Changes before and after 4‐AP 100 × 10^‐6^ m administration, (a) total number of firings, (b) number of active electrodes, (c) normalized average firing frequency per active electrode.

The electrical activity of human cerebral organoids prepared from iPS cells derived from Rett syndrome patients was measured using a HD‐CMOS‐MEA. The spontaneous activities of organoids cultured for 4 months were measured before and after administration of 4‐AP 100 × 10^‐6^ m. **Figure** **11**A shows a heat map of the number of firings detected in the spontaneous firings of organoids for 153.6 s. The area where organoids adhered to the electrodes occupied 14 612 electrodes. Before 4‐AP administration, firing was detected from 4605 electrodes out of 14 612 electrodes, and activity could be acquired from 31.5% of the electrode area. After 4‐AP administration, active electrodes increased to 7260 electrodes (49.7%) (Movie S4, Supporting Information). Figure 11B shows a raster plot of 14612 electrodes and a histogram of the number of firings (bin = 100 ms). Stripped black lines in raster plots represent network burst firings. Figure 11C shows a 3D raster plot of the organoid activity for 3 s indicated by the red line in Figure 11B. As a result of detecting the number of firing electrodes per 5 ms, the maximum number of electrodes before 4‐AP administration was 283 electrodes (average 31.8 electrodes). At the same time, the maximum number after 4‐AP administration was 339 electrodes (average 45.5 electrodes). An increase in the maximum number of electrodes indicates an enhancement of network‐wide synchrony within the burst firings. An increase in the average number of electrodes suggests an enhancement of local synchronized firing at the single‐cell level. (Figure 11C). Figure 11D shows the total number of firings, number of active electrodes, and average firing frequency per electrode before and after 4‐AP administration (n = 4 samples). 4‐AP increased the total number of firings by 232.0% (vehicle: 270540.8 ± 60282.8 spikes; 4‐AP: 627545.3 ± 114365.0 spikes) and number of active electrodes by 156.8% (vehicle: 6908.8 ± 2548.7 electrodes; 4‐AP: 10835.8 ± 3647.8 electrodes). The average firing frequency per electrode increased by 158.9% (vehicle: 0.495 ± 0.104 Hz, 4‐AP: 0.786 ± 0.292 Hz). In summary, we found that it is possible to acquire a wide range of spontaneous activities of human brain organoids, detect changes in detailed network burst patterns, and detect changes due to drug administration.

## 3 Discussion

Using the HD‐CMOS‐MEA, which can sense a wide area of 5.5 mm × 5.9 mm with 236880 electrodes (Figure 1), we recorded the propagation between the hippocampus and cerebral cortex with a sufficient S/N ratio (Figure 2). In the case of mouse brain slices, the hippocampus and cerebral cortex can be measured simultaneously, and able to analyze the propagation between brain regions in detail. As shown in the voltage color map in Figure 2E and Movie S1 (Supporting Information), it is a feature of our HD‐CMOS‐MEA that the tissue structure of the brain slice can be understood from the LFP sink and source. We found a relationship between sink and source in the apical dendrites and dorsal dendrite regions of the hippocampal pyramidal neuron (Figure 2C‐g). However, sink and source appeared depending on the firing pattern, even in the same dendrite region. It has been reported that the sink and source patterns vary depending on the input.^[^ ^15^ ^]^ By using our HD‐CMOS‐MEA, it will be possible to investigate the diversity of sinks and sources extensively.

The signal amplitudes and SNRs depending on the brain region were observed (Figure 2C‐g). The intensity of the signal depends on (1) the number of neurons firing synchronously via synaptic propagation, and (2) adhesion between the electrode and tissue. Therefore, it was considered that the hippocampus has a stronger signal intensity than the cerebral cortex since a larger number of neurons participating in synchronous activity were maintained at the hippocampus circuit structure during slice preparation. Synchronous activity in the hippocampus and cortex was observed (Figure 2E). A report by Cappaert et al. showed that the entorhinal cortex(EC)to hippocampus CA1 pathway and the EC to CA3 pathway were observed from the electrical activity of brain slices, and it is thought that the hippocampal‐cortico synchronous activity obtained in this study is the same result.^[^ ^16^ ^]^ However, for quantitative analysis, it is necessary to improve the system for stable long‐term measurement in further works. Since the propagation of EC and hippocampal is observed in epilepsy, detailed time‐series pattern measurement will advance research on the timing of EC‐hippocampal synchronous activity. Additionally, through disease models and pharmacological tests, it is expected that epilepsy will be understood and applied to drug discovery and development.

Cognitive function in the brain involves synchronizing neural activity in the gamma frequency band. Cognitive deficits in schizophrenia are associated with abnormalities in the gamma‐oscillation of cortical circuits.^[^ ^17^ ^]^ Oscillations of high‐frequency activity, known as SPW‐R, are thought to facilitate communication between the hippocampus and the neocortex and promote long‐term memory storage in cortical networks through strengthening intracortical connections.^[^ ^13^ ^]^ It is also known that SPW‐R transmission from the hippocampal dorsal CA1 to the granular retrosplenial cortex (gRSC) conducts via the subiculum.^[^ ^14^ ^]^ SPW‐R is also a marker of epilepsy, such as Dravet syndrome, and in a model of schizophrenia, SPW‐R frequency, power, and frequency changes have been reported.^[^ ^18^ ^]^ In our HD‐CMOS‐MEA, we succeeded in capturing the propagation pattern of *γ*‐oscillation, SPW‐R propagation in the hippocampus, and propagation to the cortex (Figure 3). Propagation analysis of frequency characteristics using our HD‐CMOS‐MEA has the characteristic of being able to analyze over a wide area, and it is thought that it will bring new understandings on cognitive functions and neurological diseases, and for evaluation of convulsive toxicity of pharmaceuticals.

We also found that the electrical activity of the hiPS cell‐derived cortical network can be analyzed on a cell‐by‐cell basis (Figures 4, 5, 6). Traditional MEA recordings also provide a means of generating electrophysiological activity from a large number of neurons. However, resolving the activity of individual neurons is challenging due to the poor spatial resolution of MEA data acquisition since the density of detectors is sparse on a cellular scale, which effectively renders each electrode a point detector. Despite this, it is possible to deconvolute the mixed signals of multiple neurons detected on each recording electrode of a MEA through the spike sorting analysis in which shape parameters of waveforms arising from a population of neurons are binned by similarity allowing for attribution of individual waveforms to a cell of origin.^[^ ^19^ ^]^ Parameters such as spike time tiling coefficient are used in inferring whether the two cells are functionally connected. Those bona fide connections thereby are considered to represent network activity among the neuron pairs.^[^ ^19^, ^20^ ^]^ However, the results of spike sorting performed in this fashion come with the caveat that there is no “ground‐truth” since it is impossible to know exactly how many individual neurons are being recorded. Signals arising from separate cells may be conflated as coming from the same point of origin or vice versa due to fluctuations in field potentials and propagation of potentials across the array.^[^ ^19a,b^ ^]^ On the other hand, the CMOS‐MEA provides high spatiotemporal‐resolution electrical recording within a large sensor area at high electrode density, which allows for the acquisition of electrophysiological recordings at a subcellular resolution.^[^ ^21^ ^]^ CMOS‐MEA detects signals with high sensitivity and allows to pack electrodes to be very close to each other. Therefore, the spontaneous activity from an individual neuron could be recorded by multiple electrodes with high signal‐to‐noise ratios. The advanced spike sorting method achieves single‐cell analysis for primary‐neuron cultures on CMOS‐MEA, and the network connectivity is detected between specified neuron pairs.^[^ ^5^, ^22^ ^]^ However, this single‐neuron analysis using CMOS‐MEA is limited for signals only recorded from a preselected area up to about 1000 electrodes, due to the circuit‐design constraints, i.e., the little available area to realize high‐performance circuits. Also, the network connection study is suboptimal compared to a whole‐sample recording. Compared to our previous report, we demonstrated single‐neuron analysis of hiPSC‐derived neuron cultures on HD‐CMOS‐MEA with soma discrimination using cosine similarity methods after recording the entire sample with over 59220 electrodes. Soma identification using cosine similarity is a method to remove spikes with different times. Since the soma regions are simultaneously active, no electrode of axonal propagation or conduction delay is supposed to be detected.

The cultured hiPSC‐derived cortical neurons exhibited a firing rate of 0.9 Hz at DIV46, a value comparable to that observed in the in vivo cortex (Figure 4A‐c). The feature of increasing the NB to 4‐AP and the feature of increasing the duration in a NB of PTX are the results reported in the conventional MEA measurement.^[^ ^7c^ ^]^ Owing to the parameters with significant differences were different between network analysis and single neuron analysis, it is considered that a more accurate analysis will be possible by analyzing them together. The Z score with other neurons calculated from the time‐series data for each neuron is also considered an effective analysis method which reflects changes in synaptic connection strength.

Connections with a Z score of >3 have a high probability that one neuron will be firing when the other neuron is firing. This suggests that the combination of neurons is in excitatory connection. Conversely, a connection with a Z score of <3 has the probability that one neuron will be firing when the other neuron's firing is low. This suggests that the combination of neurons is in inhibitory connection. 4‐AP acts as a K + channel blocker and Ca channel agonist, increasing the number of firings not only in excitatory neurons and inhibitory neurons, so it is thought that a significant increase in the rate of excitability connection was not observed (Figures 5 and 6). PTX, on the other hand, increased the rate of excitability connection by blocking GABA‐A receptors. It has been reported that PTX increases the rate of excitability connection and changes to repetitive excitatory activity.^[^ ^23^ ^]^ Since the increase in repetitive excitatory activity is reflected in the increased Z score, it is thought that the Z score increased due to the increase in excitatory propagation (Figures 5 and 6). The decrease in ERC in Figure 6 reflects inhibitory connections. However, it is challenging to detect inhibitory connection as a numerical value by evaluating inhibitory connection only by Z score (Figure 5) since synchronous burst firing occurs. If only inhibitory connection is to be quantified, focusing on firings other than synchronous bursts may be effective.

Combining hiPSC‐derived neurons with large‐scale electrophysiological techniques, such as CMOS‐MEAs, is thought to provide a powerful and scalable platform for neuropathy study.^[^ ^7^, ^24^ ^]^ However, such research is rarely reported, partly due to the lack of suitable metrics in downstream data analysis.^[^ ^25^ ^]^ It is reported that, retigabine, an anticonvulsant, reduces neural activity in a dose‐dependent manner from hiPSC‐derived neuron cultures on CMOS‐MEA, based on traditional network burst analysis.^[^ ^7v^ ^]^ However, the number and density of selected electrodes could be largely influenced by the result. A comparably large number of electrodes (1020 electrodes simultaneously in our report) could increase the reliability of extracted metrics. In this meaning, approaching the present study, a large‐scale single‐neuron analysis based on the big data acquisition by our HD‐CMOS‐MEA and the analysis of synaptic connection using Z score showed considerable potential to extract highly reproducible metrics for accurate assessment of drug effects on hiPSC‐derived neuron cultures.

DRG neurons are composed of several types of neurons and glial cells with large heterogeneity. Traditionally, DRGs are categorized into three subtypes based on their morphology such as soma size and myelinated/unmyelinated fibers.^[^ ^26^ ^]^ With the development of single‐cell leveled analysis, the classification of DRG neurons was further subdivided into 17 clusters based on different gene expression patterns.^[^ ^27^ ^]^ However, one limitation of such large‐scale single neuron classification based on similar transcriptomic profiles is the lack of direct functional correlates.^[^ ^28^ ^]^ In the present study, we provide a strategy in identifying DRG single‐cell classification based on neural electric activity, which has not been reported before. By using HD‐CMOS‐MEA, over 300 individual DRG neurons could be monitored simultaneously, which enable large‐scale analysis and up to 10 subtypes of DRG neurons were classified by the frequency of spontaneous activity. Interestingly, we observed DRG neurons with a relatively high firing rate (close to 100 Hz), but at a low population (1% of whole identified DRG neurons). Neurons with high firing frequency were well identified in the cortical areas, but never in the peripheral nerves.^[^ ^29^ ^]^ A critical finding of this study is that we observed heterogeneous responses to TRP agonists in different subtypes of DRG neurons, which means that it is possible to identify particular functional responses in the specific DRG subtype. It is important to clarify the relationship between the spontaneous activity pattern of single neurons and the response to drug administration, which is expected to be an effective evaluation method for peripheral neural response mechanisms to pain‐related compounds. Furthermore, we would also like to classify DRG neuron subtypes based on morphology and single‐cell transcriptomic expression in the future. For which purpose, it is necessary to investigate in detail the consistency of the cell body centroid obtained from electrical measurement and morphometry observation. This will also clarify the relationship between the previously reported 17 clusters and the electrical activity pattern of a single DRG neuron. Therefore, it is possible, in principle, to characterize the functional role of a specific gene isoform/ion channel/receptor in the relevant neuronal circuit to peripheral pain or toxicity.

Paclitaxel is widely used in treating some tumors, however it is known to cause peripheral neuropathy.^[^ ^30^ ^]^ A vitro study confirmed paclitaxel toxicity in cultured DRG neurons after 1× 10^‐6^ m exposure for 24 h.^[^ ^31^ ^]^ Using rat models, it is confirmed that TRPV1 upregulation contributes to paclitaxel‐induced peripheral neuropathy.^[^ ^32^ ^]^ Li's group showed that 12.5 × 10^‐6^ m paclitaxel treatment over 1 d significantly sensitized the TRPV1 response to capsaicin burning in cultured DRG neurons.^[^ ^33^ ^]^ In the present study, we have shown, similar to our previous report, the increase of TRPV1 sensitivity to capsaicin after paclitaxel treatment (at a lower concentration), possibly due to the high spatiotemporal resolution as proposed by our HD‐CMOS‐MEA, which allows the detecting of DRG neuronal activity at a single‐cell level.^[^ ^8a^ ^]^ Paclitaxel could also potentiate mechanosensor‐mediated signal transduction, such as the piezo2 channel, in cultured cells.^[^ ^34^ ^]^ Piezo2 has been confirmed to locate in rat peripheral sensory neurons, and increasing expression level of piezo2 is related to mechanical sensitivity in multiple pain conditions such as touch sensation or innoxious mechanic pain in rat models.^[^ ^35^ ^]^ Therefore, it is considered that paclitaxel administration could advance mechanosensitive channels such as piezo2 in cultured DRG neurons, which induced higher neuronal sensitivity to vehicle administration, as observed in the present study.

Axonal parameters, such as axonal extension length and signal propagation velocity, can be used in investigating axonal dysfunction related to neurodegenerative diseases and drug testing.^[^ ^36^ ^]^ In previous studies, microfluidic devices were used to allow axon elongation and signal propagation along axon was recorded by patch clamp or MEA.^[^ ^37^ ^]^ However, due to the very low amplitude of the axonal signals, these studies relied on repetitive electrical stimulation of an axonal segment to track signal propagation. CMOS‐MEAs provide high spatial resolution, and therefore help trigger spontaneous signal propagation along the axonal arbors, such as in cultured primary hippocampus neurons and hiPSC‐derived cortical neurons.^[^ ^5^, ^7^, ^38^ ^]^ Shimba's group has also recorded signal conduction along myelinated DRG fibers under optical stimulation using a CMOS‐MEA.^[^ ^39^ ^]^ We recorded spontaneous axonal conduction dynamics in cultured DRG neurons in the present study using HD‐CMOS‐MEA (Movie S5, Supporting Information). With the high spatiotemporal resolution provided by HD‐CMOS‐MEA, it has not necessary to compartmentalize DRG cultures using microfluidic, but detailed information including axonal pathway map and latency time, was directly shown. Since most peripheral nerves (particularly nociceptive C‐fibers) are unmyelinated, this result is also considered to recapitulate in vivo mechanisms. Exactly, potential conduction velocities were found to be 1–2 m s^‐1^ when measuring unmyelinated C‐fibers in rats, which shows the same order as our result.^[^ ^40^ ^]^

Interestingly, a backward signal propagation initiated from the distal part of the axon was observed in DRG neurons. Such bidirectional signal conduction was also recently reported in the organotypic DRG explants.^[^ ^41^ ^]^ However, to our knowledge, no in vivo evidence exists of signal backpropagation in peripheral nerves, and the functional implications of such signal backpropagation in DRG neurons are still unclear.

Vincristine is a wildly used chemotherapeutic drug to treat a variety of cancers, but could induce serious peripheral neuropathy, which is the main factor restricting its clinical application.^[^ ^42^ ^]^ The present study observed an initial increase in propagation velocity after 2 h vincristine treatment. It is reported that anticancer drugs could increase poration of membranes which leads to hyperexcitability at low, pretoxic concentrations, that can explain the increased activity while the propagation velocity was reduced after 24 h and returned to the same level as before the vincristine treatment, while the propagation distance was further reduced.^[^ ^43^ ^]^ Indeed, several groups have reported that vincristine treatment did not influence nerve conduction velocity but could induce axonal degeneration in clinical studies.^[^ ^44^ ^]^ This phenomenon corresponds to our findings. However, vincristine treatment was performed at a significantly lower concentration here than previous studies. It should also be noticed that several groups have shown conduction velocity reduction under high‐dose vincristine treatment, probably due to the asymmetric soma size/diameter in different DRG subtypes, as mentioned above.^[^ ^45^ ^]^ We would like to explore the relationship between the conduction velocity and the concentration or treatment time of vincristine in the future, to further understand the mechanisms related to chemotherapy‐induced peripheral neuropathy. Axonal conduction velocity measurement with HD‐CMOS‐MEA has the characteristic of being able to capture functional changes at low doses.

Human brain organoids serve as samples for the elucidation of neurological diseases, drug screening, and drug toxicity prediction research from the perspective of in vitro to in vivo extrapolation (IVIVE). The functional evaluation of brain organoids is mainly performed by MEA measurement, and it is reported that the electrophysiological characteristics of brain organoids which were obtained by MEA measurement are correlated with the maturation of neural networks over time revealed by scRNA‐seq., demonstrating the advantages of having a 3D structure and the effectiveness of MEA measurement.^[^ ^9f^ ^]^ In addition, drug screening using the electrophysiological characteristics of brain organoids obtained by MEA measurement has been proposed.^[^ ^46^ ^]^ It has been reported that the promising effects of NitroSynapsin for the treatment of AD were captured using human brain organoids.^[^ ^47^ ^]^ These reports indicate that human brain organoids are excellent samples for drug screening and neurological disease research in extrapolation to Vivo. By analyzing low‐frequency signals obtained by MEA measurement of cerebral organoids, we evaluated the anticonvulsant properties of drugs and the effects of antiepileptic drugs. We reported that the results were effective for in vivo extrapolation.^[^ ^48^ ^]^ Regarding brain organoid measurement using CMOS‐MEA, it has been reported that CMOS‐MEA measurements with 1024 recording electrodes can evaluate the effects of drugs on large‐scale and local neural networks.^[^ ^49^ ^]^ However, the activity data of brain organoids obtained by CMOS‐MEA measurement is a huge amount of big data, and the analysis method is highly complicated. It is expected that the establishment of an analysis method will be a breakthrough for further advancements. In this study, we measured electrical activity at the single‐cell level with 7260 electrodes over a wide range. In addition, detecting a phenomenon in which activity frequency increases in 4‐AP administration showed that it is an assessment method that can be used to predict the seizure liability and drug efficacy of pharmaceuticals. The increased activity frequency for 4‐AP was also observed in hiPSC‐derived neurons and brain slices, and showed a similar tendency.^[^ ^7^, ^50^ ^]^ An expansion of the synchronous activity range (Figure 11C) was observed, and it was found to have a feature that allows analysis of drug response based on more detailed propagation patterns. Although the correlation of electrical activity with the structure of brain organoids is required to be analyzed in future, the HD‐CMOS‐MEA measurement of brain organoids may be helpful for drug screening and functional evaluation of human brain diseases using diseased brain organoids.

We present a detailed and single‐cell‐level neural activity analysis platform for brain slices, human iPS cell‐derived cortical networks, peripheral neurons, and human brain organoids. The detailed analysis of neural activity at the single‐cell level using our CMOS‐MEA provides a new platform for understanding the basic mechanisms of brain circuits in vitro and ex vivo, as well as in vitro to in vivo extrapolation, and for exploring human neurological diseases, drug discovery, and compound toxicity assessment.

## 4 Experimental Section

### CMOS‐MEA System

The HD‐CMOS‐MEA features 236880 platinum (bare Pt) electrodes with a size of 11.22 × 11.22 µm and a separation of 0.25 µm, and 33840 readout channels operating at 70 kHz with a noise level of 9.43 ± 1.77 µVrms. The 236880 electrodes are horizontally divided into 14 blocks, with 33840 electrodes arranged per block. The arbitrarily selected blocks are sequentially accessed to read out 14‐bit signals from the electrodes. Any block can be simultaneously measured by changing the sampling frequency.

Additionally, the MEA enables simultaneous measurement of a wide area of 5.51 × 5.91 mm^2^. This MEA employs a disaggregated differential amplifier, one‐sided feedback, and an auto‐zero circuit to reduce the pixel size and suppress noise. Moreover, it integrates single‐slope ADCs, a 4.752 Gbps per ch output interface, and a stacked device structure to enhance the readout speed. CoaXpress V1.1 is used as an interface between the PC and device, and data is captured from the device to PC at a speed of 2.5 GB per s.

### Brain Slices

C57BL/6NCrSlc mice (Six weeks old) were obtained from Japan SLC. Inc. Slices of mice brain were prepared as 300 µm using a slicer (NeoLinearSlicer MT, Dosaka EM). The study was approved by Tohoku Institute of Technology Animal Care and User Committee (approval number: 2020‐01). Brain slices were plated on CMOS‐MEA supplemented with 0.05% polyethyleneimine (Sigma), incubated for 1 h, then washed with DW and dried overnight. During the experiments, the CMOS‐MEA were perfused with artificial cerebrospinal fluid (in [× 10^‐3^ m]: NaCl (KCH5696, FUJIFILM) 124, KCl (160‐03555, Wako) 3, NaH_2_PO_4_ (169‐04245, Wako) 1.25, MgSO_4_ (131‐00405, Wako) 0.1, Glucose (044‐00695, Wako) 10, NaHCO_3_(191‐01305, Wako) 26, CaCl_2_(039‐00475, Wako) 3.0 with carbogen (95% O_2_, 5% CO_2_) at a flow rate of 1.5 mL min^‐1^ using a perfusion device [(Gilson, Inc.) Mini Pulse Pump III MP‐4]. The local field potential (LFP) of each brain region was measured at 1 kHz sampling rate with 236880 electrodes simultaneously.

### Culture of Human iPSC‐Derived Cortical Neurons

Before cell seeding, the surfaces of Sony CMOS‐MEA plates were coated as follows: collagen type I‐C solution (637‐00773, Nitta Gelatin) was added and incubated for 1 h, then the collagen was washed off with DW; poly‐D‐lysine solution (P7405, Sigma‐Aldrich) was then added and plates were incubated for 1 h. The poly‐D‐lysine was removed, and plates were washed with DW and dried overnight. iMatrix 511 laminin solution (892019, Matrixome) was added just before seeding the cells, plates were incubated for 1 h, then laminin‐511 was removed. Cryopreserved hiPSC‐derived cortical neurons (XCL‐1 Neurons, XCell Science) were thawed and suspended in Neuron Medium (XCS‐NM‐001‐M100‐1P, XCell Science). For dispersed culture, approximately 7.0 × 10^4^ cells (8.0 × 10^5^ cells cm^‐2^) in 15 µL neuron medium were seeded directly in the middle of the Sony CMOS‐MEA plate at the location of the electrode array. After 30 min, 1 mL of neural maturation basal medium (NM‐001‐BM100, XCell Science Inc., USA) supplemented with neuron maturation supplement A (NM‐001‐SA100, XCell Science Inc., USA) and 100 U mL^‐1^ penicillin/streptomycin (168‐23191, Wako) was applied. After one week, the medium was replaced with 1 mL of BrainPhys neuronal medium containing SM1 neuronal supplement (ST‐05792, STEMCELL technologies) and Human iPSC‐derived mature astrocytes (XCL‐1 mature astrocytes, AR‐001‐1 V, XCell Science) were seeded at approximately 7.0 × 10^4^ cells in the Sony CMOS‐MEA plate. Following this, half the volume of the medium was replaced twice per week.

### Culture of DRG Neurons

DRG neurons were harvested and cultured as described previously.^[^ ^51^ ^]^ The study was approved by Tohoku Institute of Technology Animal Care and User Committee (approval number: 2020‐01). Briefly, DRG neurons were collected from a 10 week old male Wistar rats. First, the rats were asphyxiated with isoflurane and then decapitated. DRGs were harvested from the vertebral column, and the sensory neurons were dissociated by mechanical agitation after incubation for 2 h with collagenase type III (CLS3, Worthington) at 37 °C. Then, the cells were washed with Hank's balanced salt solution and further dissociated with trypsin type I (T8003, Sigma‐Aldrich). After cell counting, approximately 5.0 × 10^4^ cells (6.0 × 10^5^ cells cm^‐2^) in 15 µL BrainPhys Neuronal Medium were seeded directly in the middle of the Sony CMOS‐MEA plate. After 30 min, 1 mL of BrainPhys neuronal medium was applied. The next day, the medium was replaced with 1 mL of serum‐free medium containing 10 µm uridine and 10 µm 2′‐deoxy‐5‐fluorouridine kept for 3 d to suppress the proliferation of glial cells. Afterward, the medium was changed back to 1 mL BrainPhys neuronal medium, and half the volume of the medium was replaced twice per week. The same method as that used for the human iPSC‐derived cortical neurons was used for the coating treatment of the seeded CMOS‐MEA plate.

### Culture of Human Cerebral Organoids

Human‐derived iPSCs (HPS3036, Disease‐specific iPS cell line derived from a patient: Rett syndrome) were obtained from the Institute of Physical and Chemical Research. Briefly, iPSCs were cultured until confluent in StemFit (AK02 N, Ajinomoto) and were collected using Gentle Cell Dissociation Reagent (ST‐07174, STEMCELL Technologies) when cells were confluent on six‐well dishes. The collected cells were centrifuged for 5 min at 800 rpm at room temperature. After discarding the supernatant, 1 mL EB seeding medium (EB formation medium added 10 × 10^‐3^ m Y‐27632) was added and the cell pellet was resuspended. iPSCs were cultured at 9.0 × 103 cells per well in 96 wells to form an embryoid body (EB) using the EB seeding medium. After 2 and 4 d, 100 µL EB seeding medium was added per well. On day five, organoids were observed and spherical form samples were selected. EB seeding medium was substituted with an induction medium (ST‐08570, from the STEMdiff Cerebral Organoid Medium Kit) and incubated for 2 d to induce cell differentiation into neuroectoderm. After differentiation, newly formed organoids were embedded in Matrigel (354 277, Corning) and cultured with an expansion medium for 3 d. The expansion medium was substituted with a maturation medium, and the organoids were incubated in an orbital shaker (COSH6, AS ONE Corporation). Organoids were maintained in the maturation medium for 2 months, and the medium replenishment was performed every 3–4 d. After 2 months, the culture medium was changed to Brain Phys (ST‐05792, STEMCELL Technologies). The CMOS‐MEA tip on which the organoids were placed was coated using the same method as that used for brain slices.

### Immunocytochemistry

Sample cultures were fixed with 4% paraformaldehyde in PBS on ice (4 °C) for 10 min. Fixed cells were incubated with 0.2% Triton‐X‐100 in PBS for 5 min, then with preblock buffer (0.05% Triton‐X‐100 and 5% goat serum in PBS) at 4 °C for 1 h, and finally with preblock buffer containing a specific primary antibody (1:1000) at 4 °C for 24 h. The primary antibodies used were rabbit anti‐MAP2 (ab281588, abcom) and mouse anti‐*β*‐tubulin III (T8578, Sigma‐Aldrich). Then, the samples were incubated with the appropriate secondary antibody (antirabbit 488 Alexa Fluor, ab150077, Abcam or antimouse 488 Alexa Fluor, ab150113, Abcam, 1:1000 in preblock buffer) for 1 h at room temperature. For hiPSC‐derived cortical neuron sample, cell nuclei were counterstained with 1 µg mL^‐1^ Hoechst 33258 (H341, DOJINDO) for 1 h at room temperature. Stained cultures were washed twice with preblock buffer (5 min per wash) and rinsed twice with PBS. The immunolabeling was visualized by using a confocal microscope (Eclipse Ni, Nikon). Image intensity was adjusted using the ImageJ software (NIH).

### Pharmacological Tests

For hiPSC‐derived cortical neuron samples, spontaneous activities were recorded before treatment and after the cumulative addition to the culture medium of one of the following compounds: 4‐AP (10 × 10^‐6^ and 30 × 10^‐6^ m; 016‐02781, Wako), picrotoxin (1 × 10^‐6^ and 10 × 10^‐6^ m; 2800471, Nacalai tesque), and AP‐5 (25 × 10^‐6^ m; ab120 003, Abcam) followed by CNQX (30 × 10^‐6^ m; 032‐23121, Wako). All chemicals were dissolved in DMSO (0.1%), used as a vehicle.

For DRG samples, spontaneous activities were recorded before and after the cumulative addition of capsaicin (030‐11353, Wako) at 1 × 10^‐9^, 10 × 10^‐9^, and 100 × 10^‐9^ m to the culture medium. Then the medium was replaced with a culture medium containing 10 × 10^‐9^ m paclitaxel (161‐28164, Wako). After 24 h culture, spontaneous activities were rerecorded before and after capsaicin treatment.

For conduction velocity analysis experiments using DRG samples, spontaneous activities were recorded before, 2 h after, and 24 h after adding 3 × 10^‐9^ m vincristine (220‐02301, Wako) to the culture medium.

In the recordings and drug administration, the cultures were kept at 37 °C under a 5% CO_2_ atmosphere.

### Extracellular Recording

Spontaneous extracellular field potentials were acquired at 37 °C under a 5% CO_2_ atmosphere using a HD‐CMOS‐MEA system (Sony semiconductor solutions).^[^ ^52^ ^]^ Extracellular action potentials from all 23 6880 electrodes were detected at the beginning of each experiment, and then only the blocks in which tissue and cell activities were visually confirmed were selected for further measurement. The sampling rate, number of measurement electrodes, and measurement time for each sample were as follows: slices acquired signals from 236 880 electrodes at a sampling rate of 1 kHz for 8 s, and hiPSC‐derived cortical neuron, DRG, and organoids acquired signals from 59 220 electrodes at a sampling rate of 5 kHz for 153.6 s. Acquired data were offset corrected by least‐squares detrending every 500 samples. Human iPSC‐derived cortical neurons and DRG neurons and organoids were 100–3000 Hz bandpass filtered using a second‐order Butterworth high‐pass filter and a second‐order Bessel low‐pass filter before spikes were detected. The measurement time was controlled by a software (Sony semiconductor solutions), and all the raw data was saved on the PC.

### Signal to Noise Ratio

The noise level of the CMOS‐MEA electrode was calculated using data from a chip containing medium measured at a sampling frequency of 5 kHz. To calculate the signal‐to‐noise (S/N) ratio for each region of the brain sections, 8 s of data were acquired at a sampling frequency of 1 kHz. Twenty‐five electrodes were selected from each region, and the S/N ratio was calculated via the noise level from the maximum amplitude value during the 8 s and 0.5 s of no activity.

### Frequency Analysis

Gamma components and the ripple component in the LFP raw data were removed by a bandpass finite impulse response (FIR) filter from 30 to 100 Hz or 150 to 250 Hz using Signal Processing Toolbox in MATLAB. They were run forward and in the reverse direction and thus, without phase distortions.

Wavelet analysis was performed using a custom‐written program in MATLAB (using function cwt in package “Wavelet Toolbox”). In brief, the raw data, *f* (*t*), were transformed as follows:

(1) $$W\;\left(b,a\right)=\frac{1}{\sqrt{a}}\;\int_{-\infty }^{\infty }f\left(t\right)G\left(\frac{t-b}{a}\right)dt$$

where *a*,b denotes the scaling factor (1/Hz) and the center location (ms) of the mother wavelet function, respectively. 1/*a* varied from 0.1 to 250 Hz. G(x) is the complex Morlet function:

(2) $$G\;\left(x\right)=\frac{1}{\sqrt{\pi F_{B}}}\;\exp\left(-\frac{x^{2}}{F_{B}}\right)\exp\left(2i\pi F_{C}x\right)$$

where FB = 5 was the frequency bandwidth or wavenumber, and FC = 1 was the center frequency.

The wavelet power spectrum, *W*(*b*, *a*), is shown. The amplitude of this transform was obtained from its absolute value and was color‐coded.

### Spike Detection

Spikes at each electrode of hiPSC‐derived cortical neuron samples were detected using a voltage threshold of ±80 µV. Spikes at each electrode of DRG samples in capsaicin response were detected using a voltage threshold of ±100 µV. The spikes at each electrode of organoid samples were detected using a voltage threshold of ±200 µV. Spike detection and soma identification were performed using MATLAB (Mathworks Natick, MA).

### Burst Analysis

Network bursts in hiPSC‐derived cortical neuron samples and organoid samples were detected using a four‐step method similar to the synchronous burst detection method in conventional MEAs. Briefly, interspike interval (ISI) is calculated from time‐stamped data from multiple neurons, and bursts are detected by setting thresholds for the ISI threshold, interburst interval threshold, and the number of spikes within a burst.^[^ ^53^ ^]^

Single neuron burst at hiPSC‐derived cortical neuron samples were detected using Poisson‐Surprise (PS) method.^[^ ^54^ ^]^ Divide the timestamp data into groups of firings with intervals shorter than the average firing interval, and extract the firing group with the maximum PS value for each firing group. Here we assume that each neuron fires according to the Poisson distribution. A threshold was set for the PS value of the extracted firing group and the number of firings within the burst, and single‐cell bursts were detected. Network burst analysis and single neuron burst analysis used MATLAB in calculating the parameters.

### Connection Analysis

The firing time‐series data of each neuron was extracted, and the number of spikes fired within 100 ms for each spike of each neuron (synchronized spike) was counted for all combinations of neurons. Next, 100 surrogate time‐series data were created by randomly rearranging the interspike intervals (ISI) calculated from the time‐series data of each neuron, and synchronized spikes were counted. To investigate changes in synaptic strength due to the compound administration, the Z score for the number of synchronous spikes was calculated from the real synchronized spikes and 100 surrogate synchronized spikes. The Z score is a process of dividing the difference between the real synchronized spikes number and the average of surrogate synchronized spikes number by the SD of the surrogate synchronized spikes number. The Z‐fraction can truly reflect the relative standard distance of a number from the average. If we convert each number into a Z score, then each Z score represents the distance or deviation from a specific number to an average in terms of SD. When the Z score of the real synchronized spike exceeded 3, the combination of neurons was recognized as an excitable connection. Conversely, when the Z score was <−3, it was recognized as an inhibitory connection.

### Cluster Analysis

Hierarchical clustering started with calculating pairwise Euclidean distances between each cell, which were defined by a histogram vector of the number of firings for 10 s after capsaicin drug administration. Objects with the smallest distances were merged in each step. The clustering method defined how to go from object to cluster level when calculating the distance between two clusters using Ward linkage.

### Statistical Analysis

Multiple group comparisons were performed using one‐way ANOVA followed by Dunnett's test or Holm's test were used to calculate the significant difference between each concentration.

## Conflict of Interest

The authors declare no conflict of interest.

## Author Contributions

I.S. designed experiments; X.H., S.N., M.S., and N.N. conducted experiments; N.M., S.N., X.H., and Y.I. analyzed the data; I.S., N.M., and X.H. wrote the manuscript; I.S. supervised the project.

## Supporting information

Supporting Information

Supplemental Movie 1

Supplemental Movie 2

Supplemental Movie 3

Supplemental Movie 4

Supplemental Movie 5

## Data Availability

The data and scripts that support the findings of this study are available from the corresponding author upon reasonable request.
