# Supplementary material for: Large‐Area Field Potential Imaging Having Single Neuron Resolution Using 236 880 Electrodes CMOS‐MEA Technology
Source: Adv Sci (Weinh). 2023 Apr 23;10(20):2207732. doi: 10.1002/advs.202207732 (PMC10369302; doi:10.1002/advs.202207732)
Supplement: Supplementary file 1 — Supporting Information [file ADVS-10-2207732-s004.pdf]

ADVANCED SCIENCE
Open Access

# Supporting Information

for *Adv. Sci.*, DOI 10.1002/advs.202207732

Large-Area Field Potential Imaging Having Single Neuron Resolution Using 236 880 Electrodes CMOS-MEA Technology

*Ikuro Suzuki*, Naoki Matsuda, Xiaobo Han, Shuhei Noji, Mikako Shibata, Nami Nagafuku and Yuto Ishibashi*

# Large-area field potential imaging having single neuron resolution using 236,880 electrodes CMOS-MEA technology

**Authors**

I. Suzuki[1]*, N. Matsuda[1], X. Han[1], S. Noji[1], M. Shibata[1], N. Nagafuku[1], Y. Ishibashi[1]

**Affiliations**

1. Department of Electronics, Graduate School of Engineering, Tohoku Institute of Technology, 35-1 Yagiyama Kasumicho, Taihaku-ku, Sendai, Miyagi, 982-8577, Japan

**[*]Corresponding author:**

Ikuro Suzuki

Tel: +81-22-305-3219

Fax: +81-22-305-3219

E-mail: i-suzuki@tohtech.ac.jp

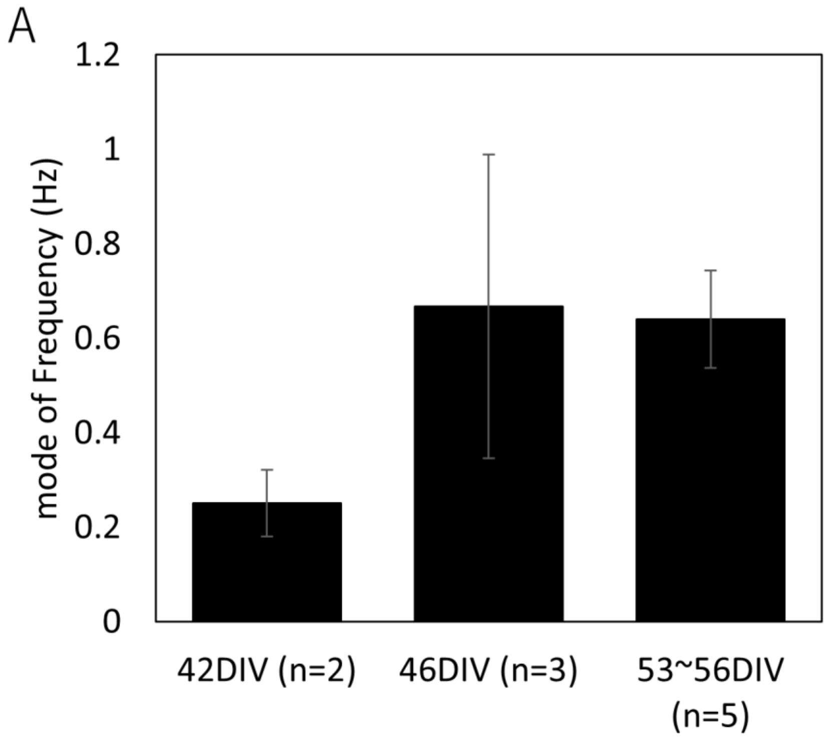


**Figure S1 The time course of the average firing frequency in human iPSC-derived cortical neurons**

**Supplementary Table S1 Representative basic statistics for each parameter of network analysis and single neuron analysis for drug administration.**

| | | | | | | | | |
|---|---|---|---|---|---|---|---|---|
| 4-AP | Concentration (μM) | 0 | 10 | 30 | Concentration (μM) | 0 | 10 | 30 |
| | TS | 83796±46049.9 | 99802.3±67229.1 | 89851.5±53563.1 | TS | 498±63.6 | 552.9±76.6 | 491.9±48.3 |
| | No. of NBs | 17.3±9.2 | 25.3±10.4 | 26.3±8.4 | No. of NBs | 17.4±5.8 | 26.5±4.9 | 25±3.9 |
| | INBI | 3389.2±1240.1 | 1836±1258.9 | 1612.4±501.9 | INBI | 3891.1±970.4 | 1785.3±239.6 | 1977.4±360.2 |
| | Duration in a NB | 1681.7±652.1 | 1594.8±639.6 | 1514.8±565.1 | Duration in a NB | 1070.9±325.9 | 1021±348.3 | 899.1±298 |
| | Spikes in a NB | 3446.3±2122.9 | 3314.6±2861.9 | 2822±2200.2 | Spikes in a NB | 20.7±2.4 | 16.7±4.9 | 13.8±3.7 |
| | MF in a NB | 470.4±235.9 | 399.7±220.9 | 358.5±174.3 | MF in a NB | 8.5±0.6 | 6.9±0.4 | 6.1±0.7 |
| | IMFI in NBs | 5059.9±1785.3 | 3418.4±1424.9 | 3160.1±1091.4 | IMFI in NBs | 4968±1260.8 | 2803.2±494.3 | 2875.8±655.2 |
| | CV of INBI | 0.468±0.26 | 0.38±0.422 | 0.14±0.029 | CV of INBI | 0.357±0.14 | 0.166±0.03 | 0.146±0.012 |
| | CV of Duration in a NB | 0.17±0.033 | 0.138±0.021 | 0.158±0.028 | CV of Duration in a NB | 0.497±0.028 | 0.376±0.043 | 0.414±0.039 |
| | CV of Spikes in a NB | 0.155±0.055 | 0.09±0.036 | 0.085±0.021 | CV of Spikes in a NB | 0.44±0.042 | 0.447±0.065 | 0.53±0.087 |
| | CV of MF in a NB | 0.143±0.055 | 0.09±0.033 | 0.078±0.041 | CV of MF in a NB | 0.333±0.021 | 0.303±0.013 | 0.309±0.009 |
| | CV of IMFI in NBs | 0.343±0.172 | 0.263±0.266 | 0.125±0.101 | CV of IMFI in NBs | 0.288±0.112 | 0.122±0.025 | 0.108±0.014 |
| PTX | Concentration (μM) | 0 | 1 | 10 | Concentration (μM) | 0 | 1 | 10 |
| | TS | 169024.3±119374.7 | 153169±114694.5 | 94716.7±59397.5 | TS | 441.3±71.8 | 395.8±68.9 | 257.5±26.4 |
| | No. of NBs | 18.3±14.2 | 16±7.5 | 13.3±1.5 | No. of NBs | 17.2±7.8 | 15.5±4.3 | 12.6±1.1 |
| | INBI | 1587±616.8 | 3898.1±3253.4 | 3872.6±1306.7 | INBI | 3506.9±1480.7 | 4750.3±1855.2 | 5133.1±753.5 |
| | Duration in a NB | 1476.4±373.5 | 1940.9±294.1 | 2021.4±457.7 | Duration in a NB | 776.2±150.2 | 1086.6±143.1 | 991.4±108.6 |
| | Spikes in a NB | 5452.6±3341.4 | 6396±4026.6 | 5006.8±3450.1 | Spikes in a NB | 15±1.2 | 17.4±1.8 | 13.1±2.3 |
| | MF in a NB | 727.7±545.2 | 777.4±622.5 | 658.3±503.9 | MF in a NB | 7.5±1.2 | 7.2±0.8 | 6.7±0.6 |
| | IMFI in NBs | 3110.8±454.3 | 5814.2±3296.8 | 5889.3±874.9 | IMFI in NBs | 4274.7±1400 | 5847.3±1930.1 | 6122.7±651.3 |
| | CV of INBI | 0.295±0.191 | 0.44±0.512 | 0.28±0.265 | CV of INBI | 0.154±0.091 | 0.42±0.264 | 0.251±0.124 |
| | CV of Duration in a NB | 0.137±0.021 | 0.19±0.07 | 0.147±0.038 | CV of Duration in a NB | 0.536±0.026 | 0.536±0.048 | 0.59±0.042 |
| | CV of Spikes in a NB | 0.187±0.13 | 0.17±0.165 | 0.097±0.072 | CV of Spikes in a NB | 0.57±0.069 | 0.449±0.064 | 0.458±0.073 |
| | CV of MF in a NB | 0.287±0.24 | 0.153±0.059 | 0.077±0.04 | CV of MF in a NB | 0.374±0.037 | 0.345±0.031 | 0.344±0.025 |
| | CV of IMFI in NBs | 0.15±0.099 | 0.337±0.402 | 0.223±0.257 | CV of IMFI in NBs | 0.105±0.062 | 0.349±0.247 | 0.196±0.125 |
| AP5+CNQX | Concentration (μM) | 0 | 25 | 30 | Concentration (μM) | 0 | 25 | 30 |
| | TS | 199533.7±133531.1 | 138860±80723.6 | 131226.3±78597.7 | TS | 577.1±98.7 | 418.7±58 | 392.4±54.2 |
| | No. of NBs | 20.3±8.5 | 4.7±6.4 | 0.7±1.2 | No. of NBs | 19.1±4.3 | 2.6±1.9 | 0.3±0.3 |
| | INBI | 2888.6±1945 | 4667.1±4126.1 | 33056.9±46749.4 | INBI | 3503.2±1108 | 11762.4±1185 | 52994.7±0 |
| | Duration in a NB | 1344±324.3 | 1120.5±900.1 | 451.4±0 | Duration in a NB | 728.3±91.7 | 520.9±187.1 | 166.3±0 |
| | Spikes in a NB | 7031.9±5606.7 | 3251.8±2818.8 | 1076.5±0 | Spikes in a NB | 20±3.3 | 7.3±2.6 | 3.5±0 |
| | MF in a NB | 997±637 | 342.3±38.4 | 264±0 | MF in a NB | 8.9±1.2 | 7±1.5 | 4.1±0 |
| | IMFI in NBs | 4242.2±1947.5 | 4968.5±4370.5 | 22283.3±38595.9 | IMFI in NBs | 4234.5±1109.4 | 12120.9±1039.2 | 53116.9±0 |
| | CV of INBI | 0.62±0.654 | 0.7±0 | N/A | CV of INBI | 0.543±0.346 | 0.209±0.171 | N/A |
| | CV of Duration in a NB | 0.183±0.076 | 0.16±0.184 | 0.05±0 | CV of Duration in a NB | 0.547±0.066 | 0.671±0.022 | 0.784±0 |
| | CV of Spikes in a NB | 0.18±0.149 | 0.15±0.198 | 0.03±0 | CV of Spikes in a NB | 0.485±0.082 | 1.196±0.359 | 0.921±0 |
| | CV of MF in a NB | 0.153±0.093 | 0.065±0.078 | N/A | CV of MF in a NB | 0.34±0.025 | 0.61±0.172 | 0.394±0 |
| | CV of IMFI in NBs | 0.45±0.554 | 0.65±0 | N/A | CV of IMFI in NBs | 0.457±0.316 | 0.199±0.163 | N/A |
